# Quinoline- and Benzoselenazole-Derived Unsymmetrical Squaraine Cyanine Dyes: Design, Synthesis, Photophysicochemical Features and Light-Triggerable Antiproliferative Effects against Breast Cancer Cell Lines

**DOI:** 10.3390/ma13112646

**Published:** 2020-06-10

**Authors:** Eurico Lima, Renato E. Boto, Diana Ferreira, José R. Fernandes, Paulo Almeida, Luis F. V. Ferreira, Eliana B. Souto, Amélia M. Silva, Lucinda V. Reis

**Affiliations:** 1 Chemistry Centre of Vila Real (CQ-VR), University of Trás-os-Montes and Alto Douro, Quinta de Prados, 5001-801 Vila Real, Portugal; eurico_lima@icloud.com (E.L.); jraf@utad.pt (J.R.F.); 2 Health Sciences Research Centre (CICS-UBI), University of Beira Interior, Av. Infante D. Henrique, 6201-001 Covilhã, Portugal; rboto@ubi.pt (R.E.B.); pjsa@ubi.pt (P.A.); 3 Institute of Bioengineering and Biosciences (iBB), Higher Technical Institute, University of Lisbon, Av. Rovisco Pais, 1049-001 Lisbon, Portugal; diana.ferreira@det.uminho.pt (D.F.); luisfilipevf@ist.utl.pt (L.F.V.F.); 4 Department of Pharmaceutical Technology, Faculty of Pharmacy, University of Coimbra, Pólo das Ciências da Saúde, Azinhaga de Santa Comba, 3000-548 Coimbra, Portugal; ebsouto@ff.uc.pt; 5 Centre of Biological Engineering (CEB), University of Minho, Campus de Gualtar, 4710-057 Braga, Portugal; 6 Department of Biology and Environment (DeBA), and Centre for Research and Technology of Agro-Environmental and Biological Sciences (CITAB-UTAD), University of Trás-os-Montes and Alto Douro, Quinta de Prados, 5001-801 Vila Real, Portugal

**Keywords:** breast cancer, phototherapeutic effects, singlet oxygen, light-stability, quinoline, benzoselenazole, unsymmetrical squaraine dyes

## Abstract

Photodynamic therapy is an innovative treatment approach broadly directed towards oncological diseases. Its applicability and efficiency are closely related to the interaction of three main components, namely a photosensitizer, light and molecular triplet oxygen, which should drive cell death. Recently, several studies have demonstrated that squaraine cyanine dyes have a set of photophysical and photochemical properties that have made of these compounds’ potential photosensitizers for this therapeutic modality. In the present research work, we describe the synthesis and characterization of four quinoline- and benzoselenazole-derived unsymmetrical squaraine cyanine dyes. Except for the precursor of aminosquaraine dyes, i.e., *O*-methylated derivative, all dyes were evaluated for their behavior and absorption capacity in different organic and aqueous solvents, their ability to form singlet oxygen, their light-stability, and in vitro phototherapeutic effects against two human breast cancer cell cultures (BT-474 and MCF-7). Regardless of the nature of the used solvents, the synthesized dyes showed intense absorption in the red and near-infrared spectral regions, despite the formation of aggregates in aqueous media. Dyes showed high light-stability against light exposure. Despite the low ability to produce singlet oxygen, aminosquaraine dyes demonstrated worthy in vitro phototherapeutic activity.

## 1. Introduction

Breast cancer is the most recurrently diagnosed cancer among women worldwide, currently accounting for 25% of cancers in women and 15% of cancer-related deaths [1]. Worldwide, the disease is considered a public health concern attributed to the significantly increased number of patients both in developed and in developing countries [2]. Breast cancer is of hormonal origin, with a heritability of 31%; however, in addition to genetic conditions, behavioral factors, related to lifestyle, age, as well as the surrounding environment, can trigger its development and progression [3,4].

Despite being a common disease, therapies with high efficacy that reduce the high mortality rate and improve patients’ life quality are not yet clinically available [5,6,7]. The development of increasingly simple, less invasive, and more efficient clinical treatments is a challenge for modern medicine [6]. There are indications that radiation-based treatments have been used for thousands of years, with the Egyptians, Indians, and Chinese being the pioneers in addressing the use of sunlight together with the dry pollen of some plants for the treatment of depigmented skin lesions, also known as vitiligo [8,9]. Currently, therapeutic modalities involving radiation have emerged with applications in several areas, such as oncology [10], ophthalmology [11], otolaryngology [12,13], neurology [14,15], gastroenterology [16], and urology [17], among others.

Photodynamic therapy is based on the uptake of a photosensitizer molecule which, upon being excited by suitable wavelength light, reacts with molecular oxygen and generates oxidizing species, also designated as reactive oxygen species, such as radicals, singlet oxygen and triplet species, specific concentrations of which lead to cell death and consequent destruction of the target tissue [18,19,20]. This therapeutic strategy is already approved or is under development for the treatment of several types of tumors, such as of the skin, esophagus, and stomach, head and neck, lung and bronchi, bladder, and of the breast [18,21]. More specifically about its potential in the treatment of breast cancer, Dougherty et al. reported that by administering hematoporphyrin derivatives followed by red light exposure, the eradication of mammary tumors in rats was achieved [22]. This latter study served as a leverage for further breast cancer studies; in addition to modernizing light sources [23,24], novel photosensitizers were designed and chemically prepared, tested in vitro [25] and in vivo [26,27], and their promising results have led to clinical trials [28]. The need to research and synthesize more second- and third-generation molecules with photosensitizing ability is due to the fact that, in the case of breast cancer, this is not considered a single disease, but a panoply of molecularly distinct pathologies [29,30], so patients are unable to respond positively with equal effectiveness with the same kind of treatment.

In search of new photosensitizing molecules, researchers working in photodynamic cancer therapy are still looking for the ideal compound that combines a set of properties; (i) easy to prepare with high chemical purity [31], (ii) the ability to reach and selectively accumulate in the target tissue [32], (iii) not cytotoxic in the absence of light but highly cytotoxic in the presence of the light of an appropriate wavelength [31], (iv) high absorption in the visible and near-infrared spectrum (650–850 nm), given that in this spectral range, the light has a higher capacity for permeating tissues [18], and (v) the ability to form reactive oxygen species [33,34]. 

To that end, several squaraine cyanine dyes, commonly named as squaraine or squarylium dyes, a family of synthetic dyes first-synthesized in the 1960s [35], have been the subject of photodynamic studies [25,36,37] given their relevant photochemical and photophysical properties [38,39,40]. These dyes are known to possess good photoconductivity, great stability under light exposure, and narrow and intense absorption bands within the tissue transparency spectral region [38,41], singlet oxygen production and high molar absorption coefficient [42]. Although generally associated with this class of dyes, these properties may vary depending on their structural changes [41,43]. The structure of these dyes is also closely related to their phototherapeutic behavior, governed by the cell line to which they are applied. Lima et al. found that the effect on photocytotoxicity on MCF-7 cell line was dependent on the number of carbons in the *N*-alkyl chains of indolenine-based symmetrical squaraine cyanine dyes [25,41]. Bis(3,5-diiodo-2,4,6-trihydroxyphenyl) squaraine, one of the first to be synthesized and tested for this purpose, has also shown significant value in the photodynamic therapy of breast cancer [44,45].

In the present paper, the synthesis of a series of quinoline- and benzoselenazole-derived unsymmetrical squaraine cyanine dyes is described, followed by their characterization and evaluation for their potential use in photodynamic therapy. Characteristics, such as their singlet oxygen formation, photostability, and aggregation properties were assessed and discussed. Their in vitro anticancer effects have been studied by determining cell viability in BT-474 (human ductal breast carcinoma) and MCF-7 (human breast adenocarcinoma derived from pleural effusion) breast cancer cell lines. The effects of concentration, irradiation and incubation time of the dyes were studied to find the ideal conditions in which the dyes exhibit photodynamic activity against these cancer cell lines. In addition, we also investigated the effects of introducing amino and methylamino groups in the dyes’ structure on the above-mentioned properties.

## 2. Materials and Methods

Starting materials for the preparation of quinoline- and benzoselenazole-derived unsymmetrical squaraine cyanine dyes, 1-iodohexane, 2-methylquinoline (**1**), squaric acid (**3**) and 2-methylbenzoselenazole (**7**), and the analytical grade solvents, were obtained from commercial sources and used as received without further purification, unless otherwise specified. Petroleum ether refers to the fraction 40–60 °C. Solvents were dried, as described in the literature [46], and freshly distilled. 3-Hexyl-2-methylbenzoselenazol-3-ium iodide (**8**) [47], 1-hexyl-2-methylquinolin-1-ium iodide (**2**) [47] and 3,4-dibutoxycyclobut-3-en-1,2-dione (**4**) [37] were prepared according to the literature procedures. Analytical thin-layer chromatography (TLC) was conducted on aluminum plates covered with 0.25 mm of silica gel (Merck, Darmstadt, Germany). Recrystallizations were carried out in solvent mixtures mentioned in each case. Melting points (M.p.) were determined in a hot plate binocular microscope apparatus (URA Technic, Porto, Portugal) and were not corrected. After melting the crystals, the possibility of decomposition was monitored by TLC; if there was a possibility of decomposition, it was indicated with the abbreviation dec. Proton and carbon nuclear magnetic resonance (^1^H and ^13^C NMR) spectra were obtained at a temperature of 298.15 K on a NMR Avance III 400 spectrometer (Bruker, Bremen, Germany) performing at 9.4 T, observing ^1^H at 400.13 MHz and ^13^C at 100.63 MHz, or on a NMR Bruker Avance III 600 spectrometer executing at 14.09 T, observing ^1^H at 600.13 MHz and ^13^C at 150.90 MHz. Dye samples were prepared in deuterochloroform or hexadeuterodimethyl sulfoxide (CDCl_3_ or DMSO-*d*_6_). Chemical shifts (δ) were reported in parts per million (ppm) relative to tetramethylsilane or residual solvent signals and the coupling constants (*J*) are reported in Hz. The proton splittings were described as singlet or broad singlet (s or br s, respectively), doublet (d), triplet or broad triplet (t or br t, respectively), broad quartet (br q), quintet or broad quintet (qt or br qt, respectively), or multiplet (m). Chemical shifts of ^13^C spectra were presented to the hundredths since rounding to the tenth causes signs of different carbons to appear at the same chemical shift. Distortionless enhancement by polarization transfer 135˚ (DEPT 135) spectra were used to make the assignments of the carbon signals. Visible to near-infrared (Vis-NIR) absorption spectra were recorded in acetonitrile (ACN), acetone (ACT), dichloromethane (DCM), dimethylformamide (DMF), dimethyl sulfoxide (DMSO), 1,4-dioxane (DXN), ethanol (EtOH), methanol (MeOH), tetrahydrofuran (THF), xylene (XLN), Dulbecco’s modified Eagle medium (DMEM), phosphate-buffered saline (PBS) and water on a Lambda 25 spectrophotometer (Perkin Elmer, Whaltam, MA, USA) using a 1 cm quartz cell (Hellma Analytics, Mülheim, Germany); λ_max_ in nanometers (nm). To determine the molar extinction coefficients (ε) of the squaraine dyes in the various solvents, five diluted dye solutions were prepared by taking aliquots of a known concentration stock solution, the absorbance intensities of each dye solution were plotted versus the sample concentration, and a linear fit was applied to determine the slope of the line. For easier comparison, the logarithm of these molar extinction coefficient values was estimated (M^−1^·cm^−1^). Infrared (IR) spectra were recorded using potassium bromide pellets on an IRAffinity-1S FTIR instrument (Shimadzu, Duisburg, Germany); ν_max_ in cm^−1^. The intensity of the bands was described as strong (s), medium (m), or weak (w). High- and low-resolution electrospray ionization time-of-flight mass spectra (HRESI- and LRESI-TOFMS) were assessed with a microTOF (focus) Bruker Daltonics spectrometer (located at CACTI, University of Vigo, Vigo, Spain), and the abbreviation [M]^+^ refers to the molecular ion peak.

BT-474 cells were acquired from Cell Lines Service (CLS, Eppelheim, Germany) and MCF-7 cells from American Type Culture Collection (ATCC, Rockville, MA, USA). Dulbecco’s modified Eagle medium, trypsin-ethylenediamine tetraacetic acid, antibiotics (penicillin and streptomycin), L-glutamine amino acid, fetal bovine serum (FBS), Alamar Blue, and other consumables used in the in vitro photobiological assays were obtained from Gibco (Alfagene, Lisbon, Portugal).

### 2.1. Chemistry

#### 2.1.1. Synthesis of 3-Butoxy-4-[(1-hexylquinolin-2(1*H*)-ylidene)methyl]-cyclobut-3-en-1,2-dione (**5**)

A mixture of **2** (5.70 g, 16.1 mmol) and **4** (3.60 g, 16.4 mmol) in ethanol (180 mL) was heated under reflux for 4 h 30 min, in the presence of triethylamine (2.45 mL, 17.6 mmol). After cooling at room temperature, the mixture was kept in an ice bath, allowing the product to precipitate. The crude product was filtered under reduced pressure, washed with petroleum ether and dried on a vacuum pump. Orange crystals. Yield 63%. M.p. 158–159 °C. IR (KBr) ν_max_: 2955 (w, CH), 2930 (w, CH), 1758 (w, C=O), 1691 (m, C=O), 1626 (w), 1526 (s, ArC=C), 1481 (s), 1446 (m), 1405 (w), 1343 (s), 1171 (s), 831 (w), 749 (w) cm^−1^. ^1^H NMR (600 MHz, CDCl_3_) δ: 8.51 (1H, br s, ArH), 7.52 (1H, t, *J* = 7.8, ArH), 7.46 (1H, d, *J* = 7.8, ArH), 7.36 (1H, d, *J* = 9.6, ArH), 7.31 (1H, d, *J* = 8.4, ArH), 7.22 (1H, t, *J* = 7.5, ArH), 5.22 (1H, s, C=CH), 4.81 (2H, t, *J* = 6.6, OCH_2_(CH_2_)_2_CH_3_), 4.06 (2H, br s, NCH_2_(CH_2_)_4_CH_3_), 1.87–1.80 (4H, m, OCH_2_CH_2_CH_2_CH_3_ + NCH_2_CH_2_(CH_2_)_3_CH_3_), 1.57–1.47 (4H, m, O(CH_2_)_2_CH_2_CH_3_ + N(CH_2_)_2_CH_2_(CH_2_)_2_CH_3_), 1.46–1.37 (4H, m, N(CH_2_)_3_(CH_2_)_2_CH_3_), 1.00 (3H, t, *J* = 7.2, O(CH_2_)_3_CH_3_), 0.95 (3H, t, *J* = 6.9, N(CH_2_)_5_CH_3_) ppm. ^13^C NMR (150.90 MHz, CDCl_3_) δ: 193.05, 186.19, 184.78, 173.64, 150.50, 139.21, 132.92 (ArCH), 131.04 (ArCH), 128.70 (ArCH), 124.11 (ArCH), 123.79, 123.30 (ArCH), 114.05 (ArCH), 85.82 (C=CH), 73.40 (OCH_2_(CH_2_)_2_CH_3_), 47.76 (NCH_2_(CH_2_)_4_CH_3_), 32.05 (CH_2_), 31.19 (CH_2_), 26.34 (CH_2_), 25.76 (CH_2_), 22.50 (CH_2_), 18.66 (CH_2_), 13.90 (CH_3_), 13.68 (CH_3_) ppm. HRESI-TOFMS *m*/*z*: 380.22202 [M+H]^+^ (C_24_H_30_NO_3_^+^, calc. 380.22202).

#### 2.1.2. Synthesis of 3-[(1-Hexylquinolin-2(1*H*)-ylidene)methyl]-4-hydroxycyclobut-3-ene-1,3-dione (**6**)

To a solution of semisquaraine dye **5** (1.24 g, 3.30 mmol) in ethanol (65 mL) at 90 °C, 0.80 mL of a 40% (*w*/*v*) sodium hydroxide aqueous solution was added, and the reaction mixture was kept for further 15 min. After cooling at room temperature, 4.10 mL of a 2 M aqueous hydrochloric acid solution was added to the mixture. Then, the reaction mixture was neutralized, and it was placed into a separating funnel that contained dichloromethane. The organic phase was separated, washed twice with cold distilled water, and dried over anhydrous sodium sulfate. The solvent was removed under reduced pressure, and the obtained residue was recrystallized from dichloromethane/petroleum ether. After the crude product was filtered under reduced pressure, the obtained residue was then washed with cold petroleum ether and dried under vacuum pressure. The resulting reddish crystals (75% yield) were used in the next reaction without further purification. 

#### 2.1.3. Synthesis of 2-[(3-Hexylbenzoselenazol-2(3*H*)-ylidene)methyl]-4-[(1-hexylquinolin-1-ium-2-yl)methylidene]-3-oxocyclobut-1-en-1-olate (**9**)

A mixture of semisquaraine dye **6** (2.40 g, 7.43 mmol) and benzoselenazole-based ammonium salt **8** (3.04 g, 7.45 mmol) in a solution of 10% of *n*-butanol/pyridine (150 mL) was heated under reflux for 4 h. After cooling at room temperature, the mixture was placed into a separating funnel that contained dichloromethane (100 mL). The organic layer was washed several times with distilled water before separation by decantation, dried with anhydrous sodium sulfate, and then the solvent was removed under reduced pressure. The resulting solid was recrystallized first from methanol/diethyl ether/petroleum ether and then from dichloromethane/diethyl ether to remove some impurities. Finally, the crystals were filtered under reduced pressure, washed with heated acetonitrile, and dried in a vacuum pump. Red crystals. Yield 33%. M.p. 280–281 °C (dec). Vis-NIR λ_max_ (ACN): 686 nm, log ε = 5.11; Vis-NIR λ_max_ (ACT): 692 nm, log ε = 5.24; Vis-NIR λ_max_ (DCM): 694 nm, log ε = 5.18; Vis-NIR λ_max_ (DMF): 689 nm, log ε = 5.04; Vis-NIR λ_max_ (DMSO): 700 nm, log ε = 5.20; Vis-NIR λ_max_ (DXN): 700 nm, log ε = 5.15; Vis-NIR λ_max_ (EtOH): 674 nm, log ε = 5.18; Vis-NIR λ_max_ (MeOH): 690 nm, log ε = 5.28; Vis-NIR λ_max_ (THF): 706 nm, log ε = 5.11; Vis-NIR λ_max_ (XLN): 733 nm, log ε = 5.27; Vis-NIR λ_max_ (DMEM): 649 nm, log ε = 4.54; Vis-NIR λ_max_ (H_2_O): 631 nm, log ε = 4.58; Vis-NIR λ_max_ (PBS): 650 nm; log ε = 4.46. IR (KBr) ν_max_: 3059 (w, ArCH), 2926 (w, CH), 1618 (m, C=O), 1588 (s, ArC=C), 1449 (s, ArC=C), 1426 (s), 1336 (s), 1289 (s), 1239 (s), 1165 (s), 1080 (s), 974 (m), 813 (w), 746 (m) cm^−1^. ^1^H NMR (600 MHz, CDCl_3_) δ: 9.28 (1H, d, *J* = 9.6, ArH), 7.55–7.49 (4H, m, ArH), 7.37 (1H, d, *J* = 9.0, ArH), 7.32 (1H, t, *J* = 7.8, ArH), 7.27 (1H, d, *J* = 8.4, ArH), 7.10 (1H, t, *J* = 7.2, ArH), 7.03 (1H, d, *J* = 8.4, ArH), 6.00 (1H, s, C=CH), 5.77 (1H, s, C=CH), 4.18 (2H, t, *J* = 7.8, NCH_2_(CH_2_)_4_CH_3_), 4.01 (2H, t, *J* = 7.2, NCH_2_(CH_2_)_4_CH_3_), 1.86 (2H, br s, NCH_2_CH_2_(CH_2_)_2_CH_3_), 1.78 (2H, qt, NCH_2_CH_2_(CH_2_)_2_CH_3_), 1.54 (2H, qt, N(CH_2_)_2_CH_2_(CH_2_)_2_CH_3_), 1.48–1.32 (10H, m, N(CH_2_)_3_(CH_2_)_2_CH_3_ + N(CH_2_)_2_(CH_2_)_3_CH_3_), 0.95–0.90 (6H, m, N(CH_2_)_5_CH_3_) ppm. ^13^C NMR (150.90 MHz, CDCl_3_) δ: 181.01, 176.91, 173.11, 159.77, 150.98, 142.72, 139.25, 133.44 (ArCH), 131.23 (ArCH), 128.79 (ArCH), 128.46, 126.79 (ArCH), 126.58 (ArCH), 125.14, 124.91 (ArCH), 124.04 (ArCH), 123.37 (ArCH), 114.49 (ArCH), 112.42 (ArCH), 92.85 (C=CH), 88.91 (C=CH), 48.21 (NCH_2_(CH_2_)_4_CH_3_), 46.65 (NCH_2_(CH_2_)_4_CH_3_), 31.41 (CH_2_), 31.37 (CH_2_), 26.91 (CH_2_), 26.67 (CH_2_), 26.50 (CH_2_), 22.44 (CH_2_), 22.58 (CH_2_), 22.48 (CH_2_), 13.94 (CH_3_), 13.93 (CH_3_) ppm. HRESI-TOFMS *m*/*z*: 586.20943 [M]^+^ (C_34_H_38_N_2_O_2_Se^+^, calc. 586.20953).

#### 2.1.4. Synthesis of 1-Hexyl-2-[3-(3-hexylbenzoselenazol-2(3*H*)-ylidenemethyl)-2-methoxy-4-oxocyclobut-2-enylidenemethyl]quinolin-1-ium trifluoromethanesulfonate (**10**)

To a solution of squaraine dye **9** (0.72 g, 1.23 mmol) in anhydrous dichloromethane (50 mL), stirred under nitrogen atmosphere at room temperature, an excess of methyl trifluoromethanesulfonate (0.42 mL, 3.69 mmol) was added. After 4 h, the reaction mixture was placed into a separating funnel, quenched with a cold 5% (*w*/*v*) aqueous sodium bicarbonate solution and then with cold distilled water. The organic layer, after separation by decantation, was dried with anhydrous sodium sulfate, and the solvent was removed under reduced pressure. The crude product was recrystallized from dichloromethane/methanol/diethyl ether. Gold greenish crystals. Yield 88%. M.p. 195–198 °C (dec). Vis-NIR λ_max_ (ACN): 636 nm, log ε = 5.82; Vis-NIR λ_max_ (ACT): 640 nm, log ε = 5.91; Vis-NIR λ_max_ (DCM): 643 nm, log ε = 5.91; Vis-NIR λ_max_ (DMF): 647 nm, log ε = 5.75; Vis-NIR λ_max_ (DMSO): 651 nm, log ε = 5.82; Vis-NIR λ_max_ (DXN): 655 nm, log ε = 5.73; Vis-NIR λ_max_ (EtOH): 641 nm, log ε = 5.86; Vis-NIR λ_max_ (MeOH): 661 nm, log ε = 5.86; Vis-NIR λ_max_ (THF): 646 nm, log ε = 5.85; Vis-NIR λ_max_ (XLN): 688 nm, log ε = 5.75. IR (KBr) ν_max_: 3064 (w, ArCH), 2929 (w, CH), 1619 (w, C=O), 1565 (w, ArC=C), 1505 (m, ArC=C), 1446 (s), 1414 (s), 1348 (s), 1315 (m), 1257 (s), 1214 (m), 1181 (m), 1148 (s), 1111 (s), 1051 (m), 1030 (m), 978 (w), 752 (w), 637 (w) cm^−1^. ^1^H NMR (400 MHz, CDCl_3_) δ: 8.74 (1H, br s, ArH), 7.90 (1H, d, *J* = 9.2, ArH), 7.78–7.73 (2H, m, ArH), 7.67 (2H, t, *J* = 8.2, ArH), 7.47 (1H, t, *J* = 7.2, ArH), 7.46 (1H, t, *J* = 7.8, ArH), 7.26–7.23 (2H, m, ArH), 6.19 (1H, s, C=CH), 5.75 (1H, s, C=CH), 4.63 (3H, s, OCH_3_), 4.44 (2H, t, *J* = 7.4 NCH_2_(CH_2_)_4_CH_3_), 4.30 (2H, t, *J* = 7.6, NCH_2_(CH_2_)_4_CH_3_), 1.89 (2H, qt, NCH_2_CH_2_(CH_2_)_2_CH_3_), 1.81 (2H, qt, NCH_2_CH_2_(CH_2_)_2_CH_3_), 1.58 (2H, qt, N(CH_2_)_2_CH_2_(CH_2_)_2_CH_3_), 1.49–1.39 (6H, m, N(CH_2_)_2_(CH_2_)_3_CH_3_), 1.36–1.31 (4H, m, N(CH_2_)_3_(CH_2_)_2_CH_3_), 0.94 (3H, t, *J* = 7.0, N(CH_2_)_5_CH_3_), 0.88 (3H, t, *J* = 7.0, N(CH_2_)_5_CH_3_) ppm. ^13^C NMR (150.90 MHz, CDCl_3_) δ: 163.91, 159.39, 156.74, 152.13, 142.16, 138.85, 137.42 (ArCH), 133.09 (ArCH), 129.56 (ArCH), 128.38, 127.71 (ArCH), 126.12 (ArCH), 125.57, 125.09 (ArCH), 124.97 (ArCH), 124.28 (ArCH) 121.92, 119.79, 116.05 (ArCH), 114.28 (ArCH), 92.84 (C=CH), 89.13 (C=CH), 61.23 (OCH_3_), 49.34 (NCH_2_(CH_2_)_4_CH_3_), 47.53 (NCH_2_(CH_2_)_4_CH_3_), 31.47 (CH_2_), 31.33 (CH_2_), 27.48 (CH_2_), 27.42 (CH_2_), 26.37 (CH_2_), 26.26 (CH_2_), 22.48 (CH_2_), 22.42 (CH_2_), 15.24 (CH_3_), 13.93 (CH_3_) ppm. LRESI-TOFMS *m*/*z*: 601.23 [M+H–CF_3_SO_3_]^+^.

#### 2.1.5. Synthesis of 2-[2-Amino-3-(3-hexylbenzoselenazol-2(3H)-ylidenemethyl)-4-oxocyclobut-2-enylidenemethyl]-1-hexylquinolin-1-ium Iodide (**11**)

To a solution of squaraine dye **10** (0.20 g, 0.27 mmol) in anhydrous dichloromethane (20 mL), under nitrogen atmosphere at room temperature, an excess of 2 M ammonia solution in methanol (0.64 mL, 1.28 mmol) was added. After 24 h of stirring at room temperature, the reaction mixture was placed into a separating funnel, and washed twice with cold distilled water. After being separated by decantation, the organic layer was dried with anhydrous sodium sulfate, and the solvent was removed under reduced pressure. The resulting product was dissolved in methanol (10 mL), and an equal volume of a 14% (*w*/*v*) aqueous potassium iodide solution was added. After 2 h of stirring at room temperature, the precipitated dye was collected by filtration under reduced pressure, washed with cold distilled water, and recrystallized from dichloromethane/methanol/diethyl ether. Dark green crystals. Yield 70%. M.p. 277–279 °C (dec). Vis-NIR λ_max_ (ACN): 658 nm, log ε = 5.83; Vis-NIR λ_max_ (ACT): 664 nm, log ε = 5.85; Vis-NIR λ_max_ (DCM): 669 nm, log ε = 5.84; Vis-NIR λ_max_ (DMF): 674 nm, log ε = 5.79; Vis-NIR λ_max_ (DMSO): 679 nm, log ε = 5.80; Vis-NIR λ_max_ (DXN): 680 nm, log ε = 5.74; Vis-NIR λ_max_ (EtOH): 667 nm, log ε = 5.84; Vis-NIR λ_max_ (MeOH): 684 nm, log ε = 5.81; Vis-NIR λ_max_ (THF): 680 nm, log ε = 5.79; Vis-NIR λ_max_ (XLN): 714 nm, log ε = 5.80; Vis-NIR λ_max_ (DMEM): 625 nm, log ε = 5.10; Vis-NIR λ_max_ (H_2_O): 608 nm, log ε = 5.32; Vis-NIR λ_max_ (PBS): 628 nm; log ε = 5.09. IR ν_max_ (KBr): 3209 (w, NH), 3102 (w, ArCH), 2929 (w, CH), 1623 (w, C=O), 1565 (w, ArC=C), 1518 (w, ArC=C), 1456 (s), 1354 (m), 1274 (s), 1251 (s), 1158 (s), 1087 (w), 1069 (w), 980 (w) cm^−1^. ^1^H NMR (600 MHz, DMSO-*d*_6_) δ: 8.95 (1H, d, *J* = 9.6, ArH), 8.81 (1H, s, NH), 8.70 (1H, s, NH), 8.04 (1H, d, *J* = 9.6, ArH), 7.94 (1H, d, *J* = 7.8, ArH), 7.85–7.81 (2H, m, ArH), 7.77 (1H, t, *J* = 7.8, ArH), 7.50–7.47 (2H, m, ArH), 7.44 (1H, t, *J* = 7.5, ArH), 7.22 (1H, t, *J* = 7.5, ArH), 6.18 (1H, s, C=CH), 5.95 (1H, s, C=CH), 4.37 (2H, br s, NCH_2_(CH_2_)_4_CH_3_), 4.16 (2H, t, *J* = 7.2, NCH_2_(CH_2_)_4_CH_3_), 1.77–1.67 (4H, m, NCH_2_CH_2_(CH_2_)_3_CH_3_ + NCH_2_CH_2_(CH_2_)_3_CH_3_), 1.54 (2H, qt, N(CH_2_)_2_CH_2_(CH_2_)_2_CH_3_), 1.43 (2H, qt, N(CH_2_)_2_CH_2_(CH_2_)_2_CH_3_), 1.39–1.28 (8H, m, N(CH_2_)_3_(CH_2_)_2_CH_3_), 0.91 (3H, t, *J* = 7.3, N(CH_2_)_5_CH_3_), 0.88 (3H, t, *J* = 6.9, N(CH_2_)_5_CH_3_) ppm. ^13^C NMR (150.90 MHz, DMSO-*d*_6_) δ: 175.06, 167.63, 162.43, 158.25, 154.81, 151.21, 142.32, 138.66, 136.10 (ArCH), 132.63 (ArCH), 129.16 (ArCH), 127.89, 127.44 (ArCH), 125.85 (ArCH), 125.38 (ArCH), 124.97, 124.25 (ArCH), 124.13 (ArCH), 116.30 (ArCH), 114.14 (ArCH), 93.89 (C=CH), 89.15 (C=CH), 48.13 (NCH_2_(CH_2_)_4_CH_3_), 46.53 (NCH_2_(CH_2_)_4_CH_3_), 31.05 (CH_2_), 30.97 (CH_2_), 26.93 (CH_2_), 26.88 (CH_2_), 25.77 (CH_2_), 25.61 (CH_2_), 22.16 (CH_2_), 22.07 (CH_2_), 13.89 (CH_3_), 13.85 (CH_3_) ppm. HRESI-TOFMS *m*/*z*: 586.23254 [M-I]^+^ (C_34_H_40_N_3_OSe^+^, calc. 586.23334).

#### 2.1.6. Synthesis of 1-Hexyl-2-[3-(3-hexylbenzoselenazol-2(3*H*)-ylidenemethyl)-2-methylamino-4-oxocyclobut-2-enylidenemethyl]-quinolin-1-ium Iodide (**12**)

To a solution of squaraine dye **10** (0.20 g, 0.27 mmol) in anhydrous dichloromethane (20 mL), under nitrogen atmosphere at room temperature, an excess of 2 M methylamine solution in tetrahydrofuran (0.64 mL, 1.28 mmol) was added. After 1 h of stirring at room temperature, the reaction mixture was placed into a separating funnel and washed twice with cold distilled water. The organic layer after separated by decantation was dried with anhydrous sodium sulfate, and the solvent was removed under reduced pressure. The resulting product was dissolved in methanol (10 mL), and an equal volume of a 14% (*w*/*v*) aqueous potassium iodide solution was added. After 2 h of stirring at room temperature, the precipitated dye was collected by filtration under reduced pressure, washed twice with cold distilled water, and recrystallized from dichloromethane/diethyl ether. Dark-purple crystals. Yield 67%. M.p. 212–214 °C. Vis-NIR λ_max_ (ACN): 671 nm, log ε = 5.76; Vis-NIR λ_max_ (ACT): 676 nm, log ε = 5.81; Vis-NIR λ_max_ (DCM): 681 nm, log ε = 5.71; Vis-NIR λ_max_ (DMF) = 686 nm, log ε = 5.70; Vis-NIR λ_max_ (DMSO): 690 nm, log ε = 5.75; Vis-NIR λ_max_ (DXN): 689 nm, log ε = 5.69; Vis-NIR λ_max_ (EtOH): 677 nm, log ε = 5.77; Vis-NIR λ_max_ (MeOH): 695 nm, log ε = 5.76; Vis-NIR λ_max_ (THF): 689 nm, log ε = 5.74; Vis-NIR λ_max_ (XLN): 718 nm, log ε = 5.72; Vis-NIR λ_max_ (DMEM): 638 nm, log ε = 5.01; Vis-NIR λ_max_ (H_2_O): 617 nm, log ε = 5.27; Vis-NIR λ_max_ (PBS): 778 nm; log ε = 5.20. IR ν_max_ (KBr): 3234 (w, NH), 3065 (w, ArCH), 2929 (w, CH), 1624 (m, C=O), 1565 (m, ArC=C), 1454 (s), 1432 (s), 1344 (s), 1255 (s), 1159 (s), 1112 (s), 1055 (w), 1028 (m), 988 (m), 747 (w), 637 (w) cm^−1^. ^1^H NMR (600 MHz, DMSO-*d*_6_) δ: 8.97 (1H, d, *J* = 9.0, ArH), 8.91–8.88 (2H, m, ArH + NH), 8.80 (1H, br q, NH), 8.09 (1H, d, *J* = 9.6, ArH), 7.97–7.94 (2H, m, ArH), 7.90 (1H, d, *J* = 7.2, ArH), 7.88–7.82 (3H, m, ArH), 7.78 (2H, br t, *J* = 7.5, Ar-H), 7.72 (1H, t, *J* = 7.5, ArH), 7.52–7.40 (6H, m, ArH), 7.24 (1H, t, *J* = 7.5, ArH), 7.19 (1H, t, *J* = 7.5, ArH) 6.15 (1H, s, C=CH), 6.07 (1H, s, C=CH), 5.94 (1H, s, C=CH), 5.85 (1H, s, C=CH), 4.36 (4H, br s, NCH_2_(CH_2_)_4_CH_3_), 4.21 (2H, br t, *J* = 7.2, NCH_2_(CH_2_)_4_CH_3_), 4.14 (2H, br t, *J* = 7.2, NCH_2_(CH_2_)_4_CH_3_), 3.30 (3H, br s, NHCH_3_), 3.29 (3H, br s, NHCH_3_), 1.77–1.71 (4H, m, NCH_2_CH_2_(CH_2_)_3_CH_3_), 1.67 (4H, br qt, NCH_2_CH_2_(CH_2_)_3_CH_3_), 1.52 (2H, br qt, N(CH_2_)_2_CH_2_(CH_2_)_2_CH_3_), 1.48 (2H, br qt, N(CH_2_)_2_CH_2_(CH_2_)_2_CH_3_), 1.39 (4H, br qt, N(CH_2_)_2_CH_2_(CH_2_)_2_CH_3_), 1.36–1.24 (16H, m, N(CH_2_)_3_(CH_2_)_2_CH_3_), 0.90–0.84 (12 H, m, N(CH_2_)_5_CH_3_) ppm. ^13^C NMR (150.90 MHz, DMSO-*d*_6_) δ: 174.59, 174.43, 165.80, 165.66, 163.51, 161.06, 157.64, 157.29, 154.74, 153.45, 151.45, 150.58, 142.37, 142.21, 138.90, 138.60, 136.67 (ArCH), 135.38 (ArCH), 132.83 (ArCH), 132.51 (ArCH), 129.25 (ArCH), 128.99 (ArCH), 128.26, 128.08, 127.54 (ArCH), 127.37 (ArCH), 125.94 (ArCH), 125.70 (ArCH), 125.67 (ArCH), 125.19 (ArCH), 124.73 (ArCH), 124.42 (ArCH), 124.15 (ArCH), 123.93 (ArCH), 121.73, 119.60, 116.53 (ArCH), 116.18 (ArCH), 114.41 (ArCH), 113.99 (ArCH), 94.44 (C=CH), 93.99 (C=CH), 89.86 (C=CH), 89.30 (C=CH), 48.36 (NCH_2_(CH_2_)_4_CH_3_), 48.26 (NCH_2_(CH_2_)_4_CH_3_), 46.72 (NCH_2_(CH_2_)_4_CH_3_), 46.07 (NCH_2_(CH_2_)_4_CH_3_), 31.05 (CH_2_), 30.97 (CH_2_), 30.89 (CH_2_), 30.81 (CH_2_), 30.28 (NHCH_3_), 30.09 (NHCH_3_), 27.08, (CH_2_), 26.98 (CH_2_), 26.60 (CH_2_), 25.76 (CH_2_), 25.72 (CH_2_), 25.70 (CH_2_), 25.60 (CH_2_), 22.17 (CH_2_), 22.08 (CH_2_), 22.01 (CH_2_), 13.90 (CH_3_), 13.86 (CH_3_), 13.83 (CH_3_), 13.81 (CH_3_) ppm. HRESI-TOFMS *m*/*z*: 600.24826 [M-I]^+^ (C_35_H_42_N_3_OSe^+^, calc. 600.24900).

### 2.2. Photostability Assessment

From dimethyl sulfoxide dye stock solutions at 1 mM, working solutions of each studied compound at a concentration of 25 µM were prepared by diluting them in the same solvent or in phosphate-buffered saline. Two hundred microliters of each diluted dye solution were added to each well of a 96-well plate, and these treatments were performed in quadruplicate. The dyes **9**–**12** were repeatedly irradiated for periods of 1 min with a light-emitting diode system, the characteristics of which are found in Section 2.4, and their absorbance was measured at a wavelength close to that of maximum absorption in dimethyl sulfoxide for 20 min using a Multiscan Go spectrophotometer microplate reader (Thermo Fisher Scientific, Vantaa, Finland). Finally, a graph was made with the variation of the normalized absorbance of the dyes as a function of the irradiation time. For comparison, the commercially known methylene blue (Merck, Darmstadt, Germany) was used, which displays an absorption wavelength close to the prepared squaraine dyes herein presented.

### 2.3. Dyes’ Singlet Oxygen Quantum Yields Determination

Singlet oxygen quantum yields of squaraine dyes **9**, **11** and **12** were measured as earlier described by our research group [48]. In summary, a system equipped with a OBB OL-401 nitrogen laser (Horiba Scientific, Piscataway, NJ, USA) exiting at 337 nm with 0.60 ns pulses and 1.1 mJ/pulse, and a detection system with an indium gallium arsenide charge-coupled device (model iDus from Andor Technology Limited, Belfast, UK) working at −60 °C coupled to a fixed spectrograph (model Shamrock 163i also from Andor). During the quantum yield determinations, long pass filters were used to exclude the radiation from reaching the LFP1100 detector (CVI Laser Optics, Albuquerque, NM, USA). Phenazine (Φ_Δ_ = 0.84) was used as a standard at an optical density of 0.6. The comparison of the total areas of the emission spectra displayed for the standard sample with those displayed by the prepared squaraine dyes, using the same optical density at the excitation wavelength, allowed the calculation of their singlet oxygen quantum yields.

### 2.4. In Vitro Photobiological Assays

BT-474 and MCF-7 breast cancer cell lines were used for the evaluation of the photocytotoxicity of squaraine cyanine dyes **9**, **11** and **12**. Cells were maintained in Dulbecco’s modified Eagle medium (Nutrient Mixture F12; 1:1) (DMEM) containing 25 mM glucose and supplemented with 10% (*v*/*v*) FBS, 2 mM L-glutamine, 100 U/mL penicillin and 100 µg/mL streptomycin, in an incubator at 37 °C in controlled humidity atmosphere containing 5% CO_2_ in the air environment. Cell culture medium was renewed every two days and cells were sub-cultured before reaching 95% of confluence [49].

For biological assays, cells from confluent cultures were detached from the bottom of the culture flasks by trypsinization, counted, and resuspended in fresh culture medium in order to obtain a cell suspension at a density of 5 × 10^4^ cells/mL. Then, 100 µL of this cell suspension was added per well of 96-well culture plates. Cells were cultured for 24 h, after which the medium was removed, and cells were incubated with squaraine dye solutions (0.1, 1.0, 5.0 and 10.0 µM) prepared in FBS-free culture medium. Since the dye stock solutions were prepared in dimethyl sulfoxide, we previously ensured that the dimethyl sulfoxide maximum concentration, in the cell viability experiments, was not higher than 3%, concentrations shown previously to be non-toxic [37]. A negative control was also carried out, exposing the cells only to the cell culture medium.

Squaraine dyes’ cytotoxicity was evaluated under absence or presence of radiation (for 7 and 14 min). For the irradiation of the cells, a self-constructed aluminum gallium indium phosphide light-emitting diode array system emitting at λ = 630.2 ± 0.8 nm with a full width of half maximum of Δλ = 15.6 ± 0.2 nm and radiant flux of P = 4.3 ± 0.5 mW. This light-emitting diode array device was placed over the 96-well plate, with the emitters facing the cells, in a way that each emitter illuminated a single well at approximately 1.5 cm of distance to the wells’ bottom. The system has a ventilation apparatus coupled to the light source to avoid the overheating of the light-emitting diode lamps and limit the influence of the heat generated by them in the in vitro experiments.

At 1 h and at 24 h after radiation treatment, cell viability was assessed by means of the Alamar Blue reduction assay. Dye aqueous solutions were replaced by a 10% (*v*/*v*) Alamar Blue solution diluted in FBS-free culture medium. Then, the absorbances at 570 and 620 nm were read approximately 5 h after the addition of Alamar Blue solution in a Multiskan EX microplate reader (MTX Labsystems, Vantaa, Finland). Cell viability was calculated by analyzing the percentage of Alamar Blue reduction as previously detailed [50] and the antiproliferative effect results were expressed as a percentage of control.

The photocytotoxicity experiments were executed in quadruplicate, and their cell viability data are stated as mean value ± standard deviation. The difference between the dye treatments and the negative control was considered statistically significant for *p*-values lower than 0.05 and were determined using Student’s *t*-test. The half inhibitory concentration values, dye concentration that reduces cell viability to 50% of control, were calculated as described [51]. All data related to biological evaluation presented in this work are illustrative of at least three independent experiments. 

## 3. Results

### 3.1. Synthesis and Photophysicochemical Characterization

#### 3.1.1. Dyes’ Synthesis Strategy

**Scheme 1:**
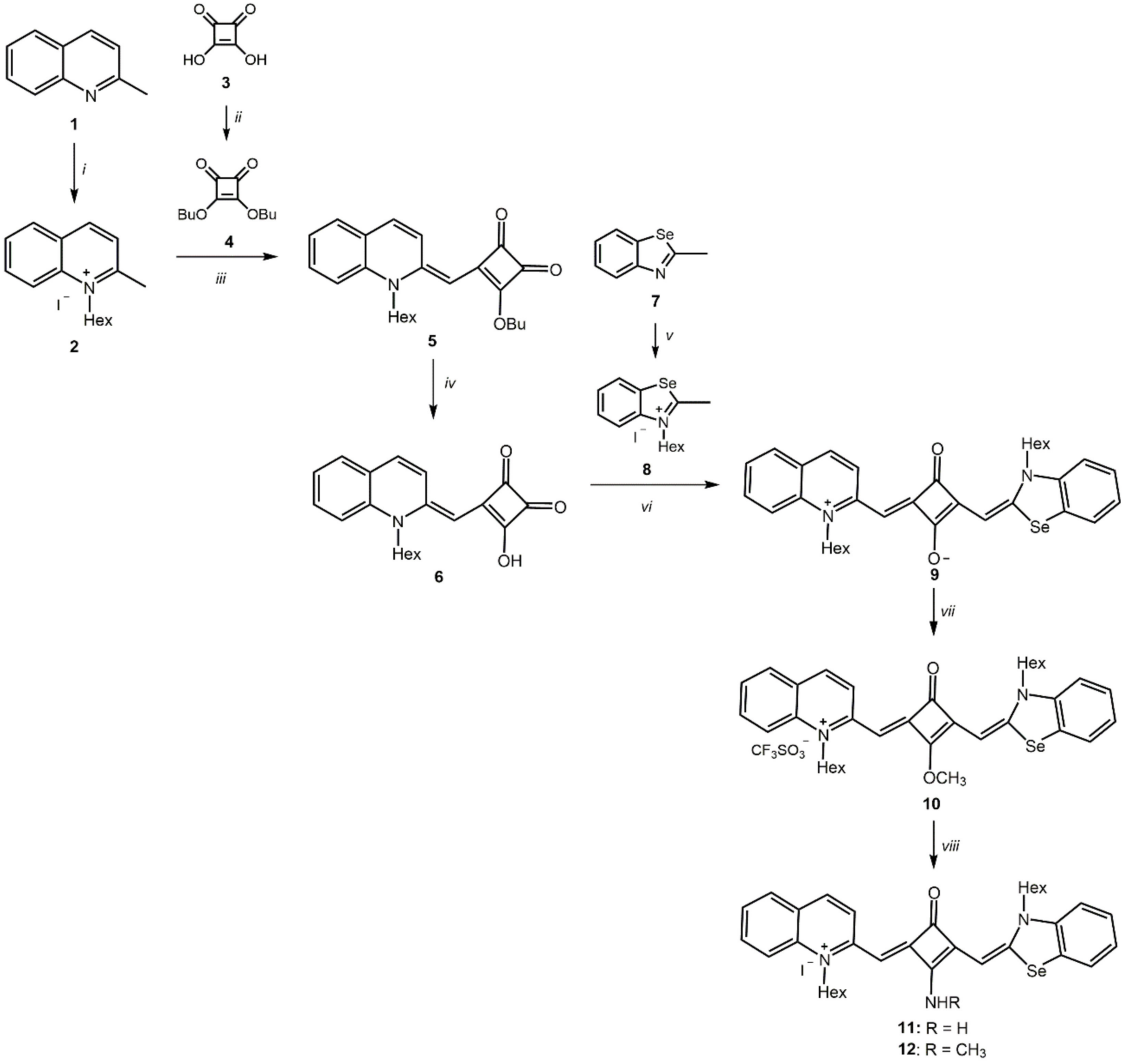
Synthetic routes of quinoline- and benzoselenazole-derived unsymmetrical squaraine cyanine dyes **9**–**12**. Reaction conditions: (*i*) 1-iodohexane, acetonitrile, reflux (η = 85%); (*ii*) *n*-butanol, reflux (η = 63%); (*iii*) trimethylamine, ethanol, reflux (η = 63%); (*iv*) 1: sodium hydroxide aqueous solution (40%), 90 °C; 2: hydrochloric acid aqueous solution (2 M) (η = 75%); (*v*) 1-iodohexane, acetonitrile, reflux (η = 83%); (*vi*) *n*-butanol/pyridine (10% *v*/*v*), reflux (η = 33%); (*vii*) methyl trifluoromethanesulfonate, dry dichloromethane, nitrogen atmosphere, room temperature (r.t.) (η = 88%); (*viii*) 1: ammonia or methylamine solution (2 M), dry dichloromethane, nitrogen atmosphere, r.t.; 2: potassium iodide aqueous solution (14%), r.t. (η = 70% and 67%, respectively).

The quinoline- and benzoselenazole-derived unsymmetrical squaraine cyanine dyes **9**–**12** were synthesized according to the multi-step route outlined in Scheme 1. The synthetic route was initiated by the conversion of 2-methylquinoline (**1**) and 2-methylbenzoselenazole (**7**) in the corresponding heterocyclic quaternary ammonium salts, 1-hexyl-2-methylquinolinium iodide (**2**) and 1-hexyl-2-methylbenzoselenazolium iodide (**8**), respectively, by alkylation with an excess of iodohexane [47]. Next, dibutyl squarate (**4**), prepared by the reaction between squaric acid (**3**) and *n*-butanol, reacted with an equimolar amount of quaternary quinoline-derived salt **2** to prepare the monosubstituted precursor **5**. After its hydrolysis by a 40% sodium hydroxide aqueous solution, and the protonation with a 2 M hydrochloric acid aqueous solution of the resulting sodium salt, the intermediate semisquaraine **6** was obtained with a moderate yield. The latter reacted with the benzoselenazole-based quaternary ammonium salt **8** in a *n*-butanol and pyridine refluxing mixture to afford the zwitterionic unsymmetrical squaraine dye **9**. The methylation reaction of this latter dye with the strong methylating agent methyl trifluoromethanesulfonate, in anhydrous conditions, allowed us to obtain the *O*-methyl ether intermediate dye **10** [39,42] with good yield. In the last step of the synthetic approach, two aminosquaraines dyes, **11** and **12**, were prepared through a nucleophilic reaction of the *O*-methyl derivative methoxy group by ammonia and methylamine solutions, respectively [25,42]. The spectroscopic characterization of these squaraine cyanine dyes, to the best of our knowledge, is described and presented here for the first time (Section 2.1 and Figures S1–S15).

#### 3.1.2. Nuclear Magnetic Resonance Particularities

In Table 1, the most relevant NMR chemical shifts of dyes **9**–**12**, the methynic protons and carbons signals are shown. For dyes **9**, **10** and **11**, the above-mentioned protons appear as two singlets of one proton each, at 6.00/5.77, 6.19/5.75 and 6.18/5.95 ppm, respectively. These two singlets that occur in the ^1^H NMR spectrum of each dye result from the fact that these specific dyes bear two different heterocycles and the chemical neighborhoods of each methynic proton, which are not precisely the same, cause two peaks with different chemical shifts. For the same reason, in the ^13^C NMR spectra of those dyes, two different peaks for methynic carbons appeared at 92.85/88.91, 92.84/89.13 and 93.89/89.15 ppm, respectively.

In the ^1^H NMR of dye **12** (Figure S13), two extra singlets can be seen in that spectral area, namely at 6.15, 6.07, 5.94 and 5.85 ppm and also, in its ^13^C NMR spectrum (Figure S14), four signals appeared at 94.40, 93.99, 89.86 and 86.30 ppm. In addition to the protons and carbons of the methynic bridges, detailed here by way of example, all other protons and carbons of squaraine dye **12** are doubled in the NMR spectra. This signals’ duplication can be explained by the difficulty in the free rotation of the bond between the nitrogen atom of *N*-methylamine substituent and the carbon of the four-membered central ring, occurring from the partial character of the double bond, which should promote the non-equivalence of chemical environments. Our research group recently observed this phenomenon in the NMR spectra of several symmetrical [25,41] and unsymmetrical methylaminosquaraine cyanine dyes [37,48].

#### 3.1.3. Absorption and Aggregation Behavior

The “phototherapeutic window” is the name assigned to the range of wavelength values between 600 and 850 nm to which candidate compounds for photodynamic therapy photosensitizers should absorb with some intensity [37,52,53]. This range of values comprises the wavelengths at which light permeates the tissues more efficiently and, at the same time, sends enough energy to the target tissue to activate the photosensitizing molecules accumulated in it [54,55]. Thus, one of the first approaches to be made in novel candidate molecules for this therapeutic strategy is the study of their absorption capacity as well the determination of their maximum absorption wavelengths, in order to know if, when the light is incurred in the living tissue, these compounds are able to capture energy from the light source, even at some depth.

For this purpose, the dyes involved in the present work were dissolved in several organic solvents (acetonitrile, acetone, dichloromethane, dimethylformamide, dimethyl sulfoxide, 1,4-dioxane, ethanol, methanol, tetrahydrofuran and xylene), to study the influence of polarity, as well as their dissolution in protic and aprotic solvents, in this photophysical property. 

**Figure 1:**
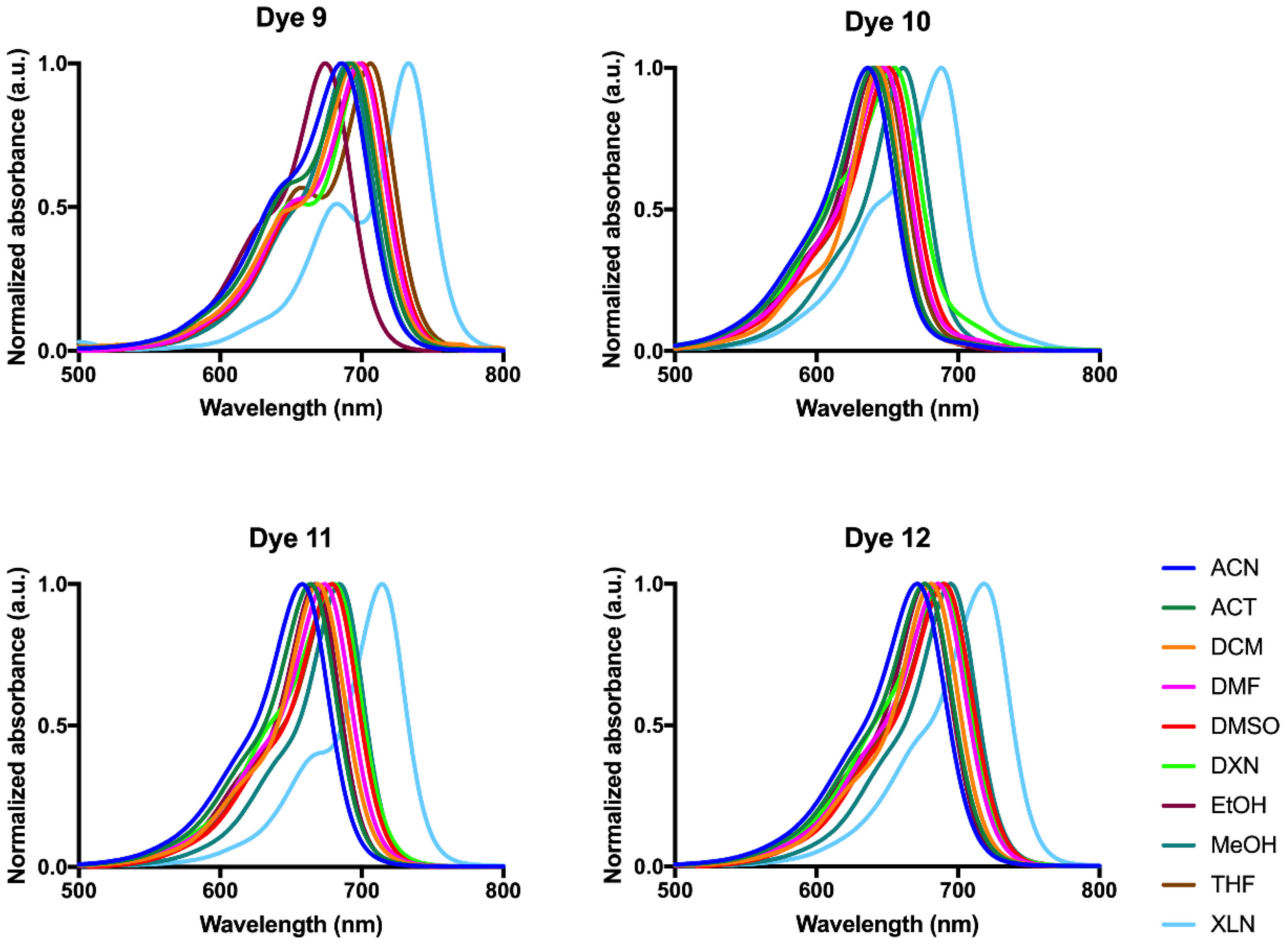
Normalized red and near-infrared absorption spectra of quinoline- and benzoselenazole-derived unsymmetrical squaraine cyanine dyes **9**–**12** acquired in acetonitrile (ACN), acetone (ACT), dichloromethane (DCM), dimethylformamide (DMF), dimethyl sulfoxide (DMSO), 1,4-dioxane (DXN), ethanol (EtOH), methanol (MeOH), tetrahydrofuran (THF) and xylene (XLN) at room temperature. Absorption is presented as arbitrary units (a.u.).

Regardless of the nature of the organic solvent, all squaraine dyes have shown to have maximum absorption wavelengths within the values at which living tissues have high transparency to light (Figure 1 and Table 2). It is also possible to verify that, in general, the synthesized dyes presented maximum absorption wavelength values very close to each other in the various organic solvents, as well as spectral bands with similar behavior, except when dissolved in xylene, the most non-polar solvent used. In the latter, the presence of two visibly distinct bands in squaraine dye **9** is noteworthy, the least intense of which corresponds not only to the typical shoulder of this compounds’ core, as previously reported in the literature, but also to the formation of H aggregates [56,57]. Due to the fact that there is no total inverse linearity between the increase in the maximum absorption wavelength of the dyes and the polarity of the organic solvents studied, and that the compounds do not exhibit marked differences in their absorption spectra, except when displayed in xylene, it appears that the synthesized compounds do not show marked solvatochromism, a behavior previously reported for dyes also derived from cyanines [58]. Whether the solvents are protic or not, it does not seem to play an important role in this photophysical parameter of the synthesized dyes, since in ethanol and methanol, both polar protic solvents, the dyes exhibit low and high maximum absorption wavelengths, respectively.

**Figure 2:**
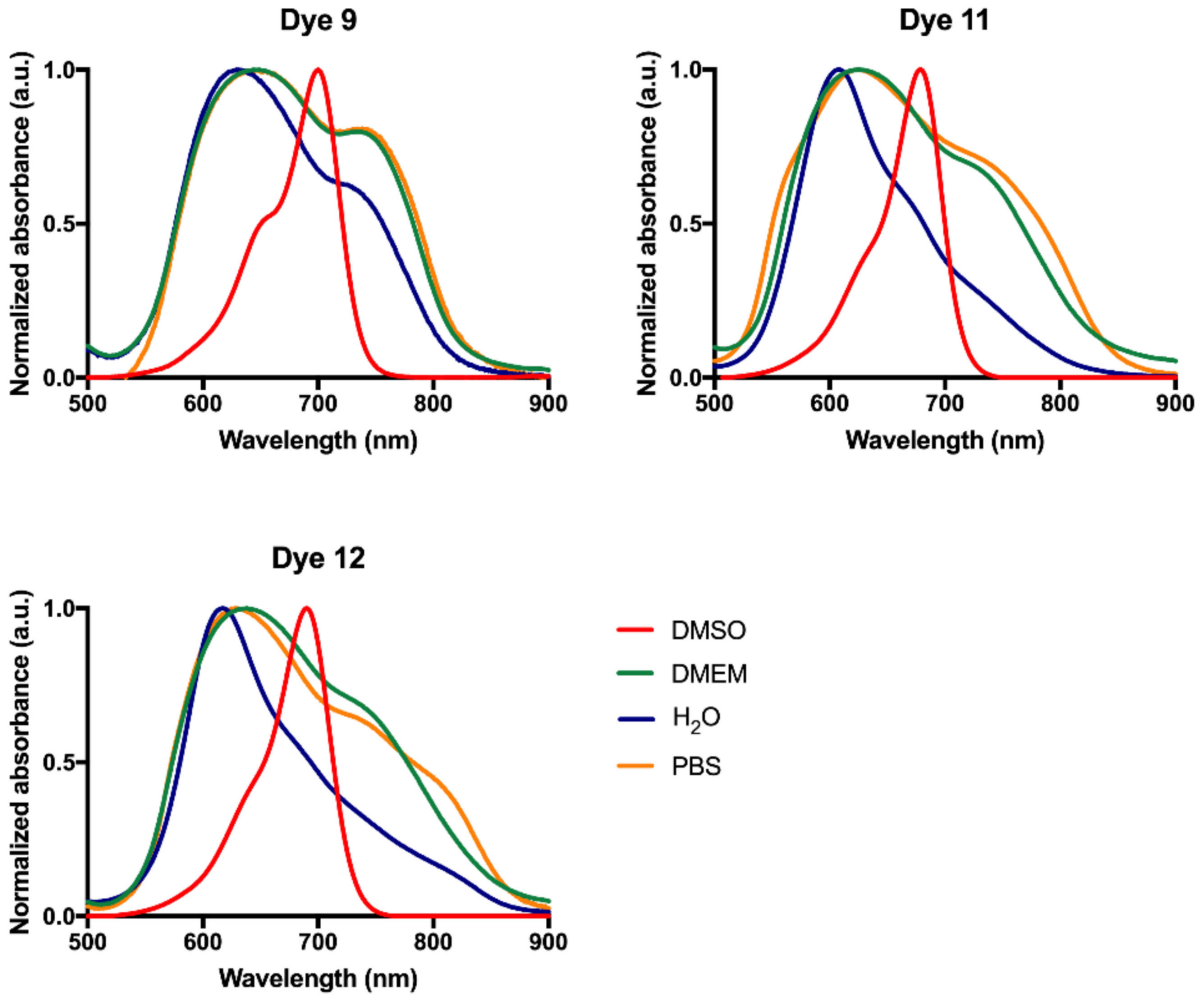
Normalized red and near-infrared absorption spectra of quinoline- and benzoselenazole-derived unsymmetrical squaraine cyanine dyes **9**, **11** and **12** acquired in dimethyl sulfoxide (DMSO), Dulbecco’s modified Eagle medium (DMEM), water and phosphate-buffered saline (PBS) at room temperature. Absorption is presented as arbitrary units (a.u.).

The combined analysis of the absorption spectra of the dyes in an organic solvent in which the dyes remain in the form of monomers (e.g., dimethyl sulfoxide) with the spectra obtained in aqueous solutions allows the assessment of the occurrence of the aggregates’ formation in these solvents (Figure 2).

The three dyes showed behavior in aqueous media utterly different from each other. For the zwitterionic squaraine dye **9**, regardless of the aqueous medium studied, there is the formation of blue-shifted aggregates, called H-aggregates. It is also observed that the tendency to form aggregates is higher than the monomeric form, since the absorbance found for the deviated band is higher than the band corresponding to the free form of the squaraine dye. Of the three aqueous solvents, the absorption spectrum in water should be highlighted, in which dye **9** is more prone to the formation of these dimers, since the band deviated to red has reduced absorbance compared to the culture medium and buffer solutions. Dye **10** was not evaluated for its aggregation character, since this dye hydrolyzes in its precursor dye **9**. Zwitterionic and aminosquaraine dyes **9** and **11**, respectively, in addition to H aggregates, the band of which is quite intense, show a tendency to form J aggregates, since a red-shifted lower intensity band at about 750 nm is presented in water. In the culture medium, the latter dye showed a profile similar to the zwitterionic dye. Interestingly, the constitution of aqueous media had a major impact on the aggregation character of aminosquaraine dyes **11** and **12**. These dyes presented very wide bands in the three aqueous media but, comparing the spectra obtained in water with those of phosphate-buffered saline and culture medium, differences are observed in the intensity with which the aggregates are formed. While in the culture medium, the tendency to form H aggregates remains, and in water a residual amount of J aggregates can be seen, in phosphate-buffered saline and culture medium, these dyes are more prone to dimerization in J aggregates, since more intense red-shifted bands are displayed at approximately 800 nm. Regardless of the aggregate character, all dyes showed proper absorption in the electromagnetic regions where the tissues are transparent to light, as well as good absorption coefficients (Figure 2 and Table 2).

#### 3.1.4. Photostable Character

The evaluation of photostability or photodegradability of photosensitizers’ candidates, such as chlorins and porphyrins, has been one of the focuses of study by some research groups [59,60,61]. This is a property of high relevance concerning their photodynamic application, since the effectiveness of their performance in the cellular environment may depend on how stable these molecules are in the presence of light, one of the three factors necessary for the occurrence of cytotoxicity production [62]. In preliminary studies, the assessment of the light-stability of photosensitizing molecules is required to verify if the cytotoxicity produced by the compound to be evaluated comes from it, or if it could be from a compound product of its degradation [62,63]. Also, as for any drug candidate, stability under light is of high significance, because, depending on this photophysicochemical property, the control made in terms of its preparation, transport and storage will be done in the most appropriate way possible to ensure that the product is as high as possible, and the administration to the patient is carried out under the suitable conditions for therapeutic use [64,65].

The photostability evaluation of the squaraine cyanine dyes **9**–**12** was made using working solutions of each dye in dimethyl sulfoxide or phosphate-buffered saline at a concentration of 25 μM, and later irradiation of these solutions using a light-emitting diode system centered at 630.2 ± 0.8 nm. The absorbance of these dyes at a wavelength close to their maximum absorption was measured in periods of 1 min for 20 min, and this has been normalized so that a comparison can be made. In addition, the methylene blue light stability, a commercial photosensitizer widely studied and already applied clinically, was also used as a standard.

**Figure 3:**
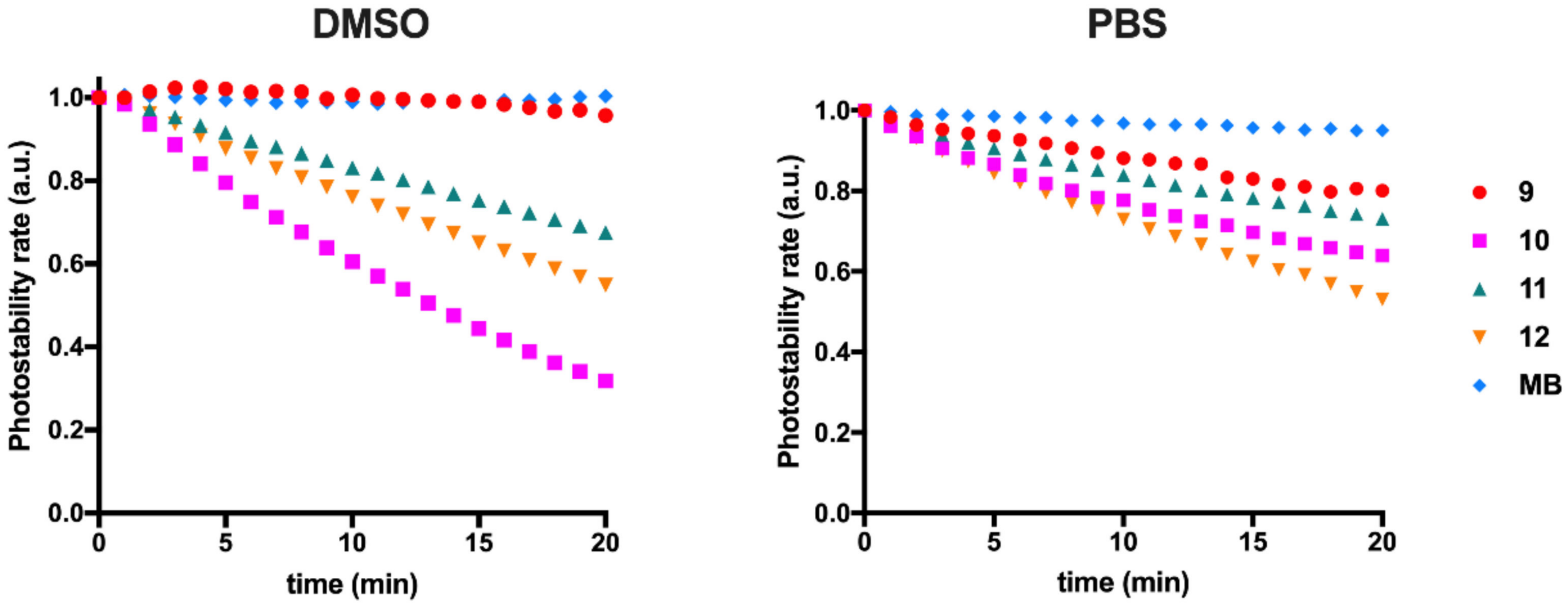
Photostability rate, in arbitrary units (a.u.), of quinoline- and benzoselenazole-derived unsymmetrical squaraine cyanine dyes **9**–**12** and methylene blue (MB) in dimethyl sulfoxide (DMSO) and phosphate-buffered saline (PBS). Dye solutions were irradiated repeatedly with a light-emitting diode system emitting at 630.2 ± 0.8 nm and radiant flux of 4.3 ± 0.5 mW for periods of 1 min for 20 min.

The variation of the substituents introduced in the four-membered central ring induced great differences regarding the light-stability of the prepared dyes, which was shown to be moderate to high (Figure 3). Compared to methylene blue, which showed high photostability to this light source, the zwitterionic dye **9** presented a similar performance. In respect to this property, the introduction of amines (dyes **11** and **12**) into the four-membered central ring led to a reduction in its light-stability. The *O*-methylated dye **10** was most degraded in the presence of this physical agent. The nature of the solvent used also influenced its performance with regard to its stability to light. While in dimethyl sulfoxide the zwitterionic dye **9** maintained excellent stability, in aqueous media this was not so evident. It is notable that the photostability became more uniform at this last solvent, with no noticeable differences between the prepared dyes. Significant improvements in its resistance to the light radiation can be seen, especially for *O*-methylated dye **10** in an aqueous environment. Methylene blue, the standard photosensitizer molecule used in the evaluation of this property, showed high stability in both solvents.

#### 3.1.5. Singlet Oxygen Formation Ability

Singlet oxygen is reported as the most important reactive oxygen species in photodynamic therapy, as it has high reactivity and produces significant cytotoxicity from the biological point of view [66,67,68], and several changes in the dye structure have been carried out to enhance the increase in its ability to produce type II reactions, a mechanism from which these species are produced [40,69]. Among the most common of the structural modifications in squaraine cyanine dyes, the introduction of heavy atoms is noteworthy, since, according to the literature, it leads to an increase in the dyes’ ability to generate type II reactions [45,70]. Despite the importance given to singlet oxygen about the potential application of novel molecules in photodynamic therapy, several studies showed that other mechanisms of action may be involved in phototherapeutic activity, namely type I reactions, in which other reactive oxygen species are produced that, despite being less responsive, are also highly toxic agents at high concentrations [37,48].

The dyes synthesized in this work showed low singlet oxygen quantum yields (Table 3), so their ability to trigger type II reactions is reduced. Even so, it is possible to verify that the introduction of amine groups in the four-membered central ring led to a slight increase in its production ability of these reactive oxygen species, since, compared to the zwitterionic compound **9**, the insertion of methylamine group (**12**) caused their quantum yield doubled. *O*-methylated derivative **10**, as it was not subject to photodynamic evaluation, for the reasons mentioned above, was not studied for this property either.

### 3.2. In Vitro Photoantiproliferative Effects

In vitro studies are undoubtedly extremely advantageous in a first approach to assess the effectiveness of novel photosensitizer candidates, given that, through the use of previously characterized cell lines, it is possible to obtain information about the intrinsic cytotoxicity of the compounds after irradiation. The phototherapeutic potential of the prepared squaraine dyes in cell cultures at first interpellations is aligned with the principle of the 3 R’s (“reduction”, “refinement” and “replacement”) [71].

As a preliminary study about the medicinal potential of dyes for the phototherapeutic treatment of breast cancer, in this research, the adherent BT-474 and MCF-7 cell lines were incubated with the synthesized compounds. Both cell lines come from the mammary gland; however, while the BT-474 cell line comes directly from its duct, MCF-7 cells are a line of epithelial cells isolated from a pleural effusion from a woman with metastatic breast cancer [29,72].

To evaluate the effect of the squaraine cyanine dyes **9**, **11** and **12** on BT-474 and on MCF-7 breast cancer cell proliferation, cells were treated at several concentrations (0.1, 1.0, 5.0 and 10.0 µM). Twenty-four hours after treatment, photodynamic irradiation was applied for 7 or 14 min on the cells using a self-designed light-emitting diode system with a red light of λ = 630.2 ± 0.8 nm and radiant flux of P = 4.3 ± 0.5 mW (see methods for details). After 1 h and after 24 h of cell contact with the irradiated dyes, squaraines’ antiproliferative effects were evaluated using the Alamar Blue colorimetric method. Negative controls related to the presence of squaraine dyes and irradiation were also performed, that are non-treated and non-irradiated cells.

**Figure 4:**
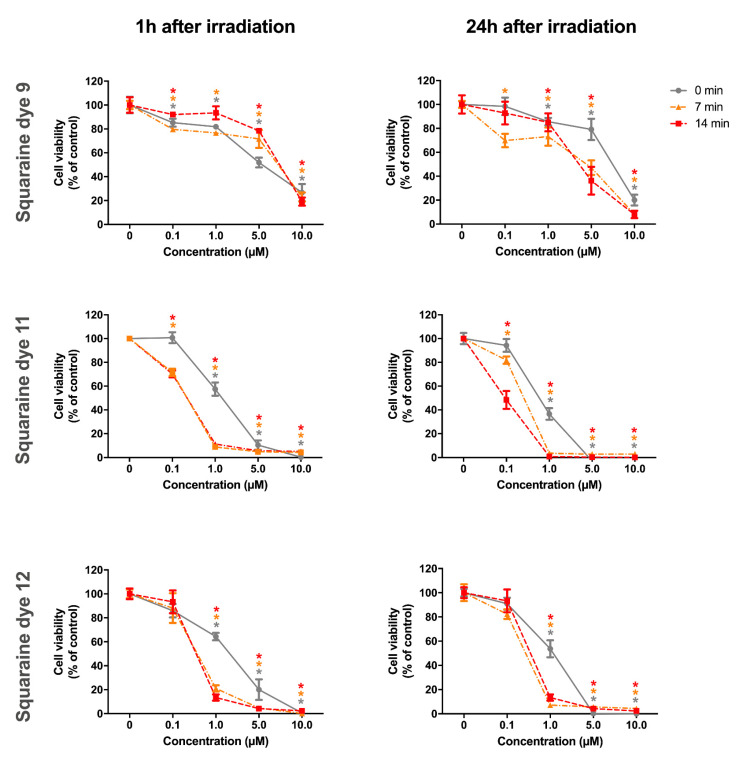
Antiproliferative effects of photodynamic treatment on BT-474 cells. BT-474 cells were exposed for 24 h to the quinoline- and benzoselenazole-derived unsymmetrical squaraine cyanine dyes **9**, **11** and **12**, at indicated concentrations, and then subjected to light-treatment using a light-emitting diode system 0, 7, or 14 min (see methods for details). After contact with the irradiated dyes for 1 h or for 24 h, cell viability was assessed. Cell viabilities are expressed as percentage of control, non-treated cells (0 μM), and presented as mean value ± standard error of three independent experiments (each one in quadruplicates). Student’s *t*-test statistical significant treatments related to the non-treated condition (*p*-value < 0.05) are evidenced by an asterisk (*) of the color relative to the irradiation time.

**Figure 5:**
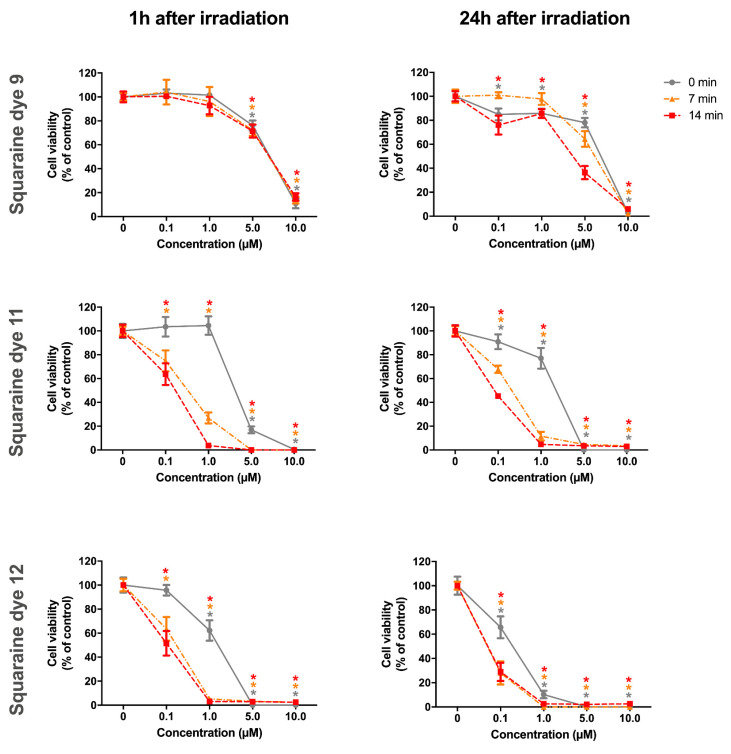
Antiproliferative effects of photodynamic treatment on MCF-7 cells. MCF-7 cells were exposed for 24 h to the quinoline- and benzoselenazole-derived unsymmetrical squaraine cyanine dyes **9**, **11** and **12**, at indicated concentrations, and then subjected to light-treatment using a light-emitting diode system 0, 7, or 14 min (see methods for details). After contact with the irradiated dyes for 1 h or for 24 h, cell viability was assessed. Cell viabilities are expressed as the percentage of control, non-treated cells (0 μM), and presented as mean value ± standard error of three independent experiments (each one in quadruplicates). Student’s *t*-test statistical significant treatments related to the non-treated condition (*p*-value < 0.05) are evidenced by an asterisk (*) of the color relative to the irradiation time.

Analyzing the activity of the squaraine dyes in the BT-474 cell line (Figure 4), it appears that aminosquaraine dyes **11** and **12** were the ones that showed the best phototherapeutic effects. Indeed, zwitterionic squaraine dye **9** exhibited higher cytotoxic activity under non-irradiated conditions compared to irradiated conditions, except for the concentration of 5.0 µM after 24 h irradiation. The decrease in toxicity observed for dye **9** under irradiated conditions, more evident at 14 min irradiation, which was not expected, can be the result of the non-effectiveness of irradiation to enhance dye toxicity in a short time (1 h), as is observed in MCF-7 cells (Figure 5). Additionally, the irradiated dye could activate multi-drug resistance transporters, or efflux pumps, in particular the ABCC3 [73]. The ABCC3 efflux pump, in the case of breast cancer cells, is associated with HER2 positive cells, which is the case of BT-747 cells [49], and has been associated to a decreased rate of drug retention and with chemoresistance [73]. On the other hand, dyes **11** and **12** presented more significant selective cytotoxicity for irradiated treatments at certain concentrations. Taking into account the Generally Recognized as Safe (GRAS) status [74,75], in which International Standardization reported that the percentage of reduction in cell viability from which compounds in in vitro tests can be considered toxic is >30% (i.e., cell viability <70%), we verify two conditions in which the dyes show a selective phototherapeutic effect: at the concentration of 0.1 µM, dye **11**, after 14 min of irradiation and 24 h of contact with irradiated dyes, and 1.0 µM of dye **12** after 1 h, at both irradiation times explored. Despite the sharp differences in cell viability of cells exposed to compound **11** bearing amino group at a concentration of 1 µM and 1 h of incubation between irradiated and non-irradiated treatments, as the condition in the dark had a decrease in cell viability of more than 30% compared to the untreated control (0 µM), no selective effects can be considered. The same is observed for the condition treated with methylamine-substituted dye **12** at the same concentration and after 24 h of exposure.

The increase in the irradiation time has been shown not to markedly increase the cytotoxicity of the studied dyes for BT-474 cells, in contrast to other aminosquaraine dyes that we recently reported to other cell lines [37], while that of the incubation period with irradiated dyes, at the lowest concentrations tested, has been shown to produce a slight decrease in cell viability.

The susceptibility of different cell lines to the dyes was verified by comparing the results obtained in the two breast cancer cell lines for the same compounds. Nevertheless, in the MCF-7 cells, once again, the aminosquaraine dyes **11** and **12** showed greater photodynamic relevance compared to their zwitterionic analogous compound **9** (Figure 5). This latter compound, only at the highest tested concentration, can produce high cytotoxicity. However, this antiproliferative effect is not selective for irradiated conditions. Interestingly, for this cell line, the aminosquaraine dyes **11** and **12** showed more intense phototherapeutic results compared to the BT-474 cell line. Dye **11**, at concentrations of 0.1 and 1.0 µM, regardless of the incubation time with irradiated dyes, showed high photoselective cytotoxicity, since, while in non-irradiated treatment, it did not cause a decrease in cell viability more significant than 30%, the irradiation for 14 min led to its occurrence (Figure 5). For dye **12**, it is already possible to verify the influence of the increased incubation period, since, both in the dark and in the light presence, the percentages of cell viability decreased noticeably. Thus, this last dye showed, although slight, selective photodynamic effects for treatments at 0.1 µM at both irradiation and incubation periods, as well as more sharply at 1.0 μM at the shortest incubation time (Figure 5).

Both irradiation times, also in the MCF-7 metastatic cell line, have been shown to produce similar effects in the cellular environment, thus the 7 min of irradiation would have been enough for the compounds to perform their maximum photodynamic activity.

The recorded half-inhibitory concentration values (which translate the pharmacological potency of a drug and the required concentration to produce a 50% reduction in cell viability) are shown in Table 4. Similarly to what was previously reported [25], the zwitterionic dye **9** presented weak and inconsistent effects on breast cancer cells, as it showed high half-inhibitory concentration values in all tested conditions. However, aminosquaraines **11** and **12** were only able to significantly reduce these values in irradiated treatments (Table 4). These differences in potency of irradiated and non-irradiated aminosquaraine dyes suggest their potential application as photosensitizers for breast cancer photodynamic therapy. The high coefficients of values’ determination (≥0.95) are suggestive of how near the achieved sigmoidal curve can be adjusted with nonlinear regression statistics for the evaluated cell lines, irradiation times, and incubation periods.

## 4. Discussion

The synthesis of unsymmetrical molecules, in the case of the dyes presented in this study, aimed at combining photophysicochemical properties which, according to some of our previous works, would be of particular interest in the formulation of novel photosensitizer candidates for photodynamic cancer therapy. The choice of quinoline and benzoselenazole heterocycles was based on the fact that the dyes with quinoline units display a much higher red shift in their absorption than other heterocycle nuclei [39], and those of benzoselenazole, as it has a heavy atom in their structure, can positively influence the production of reactive oxygen species by the so-called “heavy atom effect” [40].

All dyes, regardless of the polarity of the organic solvents studied, displayed narrow and intense absorption bands in the red and near-infrared regions, one of the most critical properties, since the light from these electromagnetic regions can better permeate living tissues, as well as making sure that energy can reach the photosensitizing molecule to be activated. Their molar absorptivity coefficients also show the high capacity of these dyes to capture energy from radiation. Compared to symmetrical quinoline-derived squaraine dyes, previously synthesized and reported by some of us [42], these unsymmetrical dyes displayed similar maximum absorption wavelengths. However, compared to the symmetrical benzoselenazole derivatives reported in the same study, the quinoline heterocycle had an impact on this photophysical character of unsymmetrical squaraine dyes, since the compounds herein presented exhibited, on average, a red shift of about 20–30 nm. 

In aqueous media, the dyes showed broader bands resulting from the aggregation in these media (Figure 2). Nevertheless, their capacity for absorbing energy remained quite high. This finding has been observed previously for other symmetrical and unsymmetrical squaraine cyanine dyes derived from other heterocycles [41,48]. The occurrence of the J-type aggregates formation, as we saw for the aminosquaraine dyes **11** and **12** in specific aqueous media, until then, was not observed by us in other squaraine dyes. We found that the constitution of aqueous media, such as the salts present in them, can have significant effects on their aggregation behavior.

Regarding the dyes’ light-stability, an approach that has been explored in our prior studies [25,41,48], the unsubstituted dye in the zwitterionic dye **9** was the one that stood out the most, as it is very stable to light (Figure 3). Aminosquaraine dyes had a worse photostable performance, since there was a more pronounced decrease in the absorbance of dyes **11** and **12** over the irradiation periods. In our last study, in which benzothiazole- and iodoquinoline-derived unsymmetrical squaraine dyes loaded with the same substituents on the squaric ring were involved [48], although the zwitterionic dye revealed less firmness than the one presented here, the aminosquaraine dyes revealed an absorbance evolution similar to that of zwitterionic. The relevance of the choice of heterocycles in this property has already been evidenced by us [76], and in this study, compared to benzothiazole-derived dyes [48], we found that the introduction of the benzoselenazole heterocycle moiety significantly improved dyes’ light stability of the zwitterionic one. Establishing a comparison between benzothiazole and benzoselenazole-derived aminosquaraine dyes, after 20 min of light exposure, benzothiazole-based dyes suffer a less marked decrease in absorbance over the irradiation time [48]. In our experience, and within this dyes’ scaffold, the dyes under study only lose to the indolenine-based squaraine dyes, since these molecules have very high stability for long periods of irradiation [25,38,41]. However, it should be noted that these comparisons may not be so linear, as light sources with different properties were used for the other studies. The comparison of the dyes’ stability in organic and aqueous solvents led us to conclude that, although the four-membered central ring unsubstituted squaraine dye **9** has a worse performance in aqueous media, and the aminosquaraine dyes **11** and **12** reduce their sensitivity to light, the zwitterionic dye **9** was the dye with the best photostability in this study. Hypothetically explanatory reasons for the differences observed in the light-stability of the synthesized dyes in organic and aqueous media are the significant reduction in dyes’ molar absorptivity coefficient in aqueous media, so these dyes will not have as much ability to absorb light energy in these solvents, as well as the tendency for the aggregates’ formation that may have increased the resistance of aminosquaraine dyes to the presence of light.

The singlet oxygen formation ability of the quinoline- and benzoselenazole-derived squaraine dyes was weak (0.03 ≤ Φ_Δ_ ≤ 0.06). These values are within those previously observed for unsymmetrical analogs [37,48]; however, it was expected that compounds with the selenium atom exhibit increased capacity for the formation of this reactive oxygen species. This speculation is explained by the fact that symmetrical dyes derived from benzoselenazole have exhibited high singlet oxygen quantum yields (0.05 ≤ Φ_Δ_ ≤ 0.31) in a previous study [77], which were determined using the same technique. Despite being reported by many as a very relevant property, studies have shown that photosensitizer candidates with low singlet oxygen production capacity can reveal very significant antitumor photodynamic activity [37,48,78].

The dyes that showed greater in vitro photodynamic activity on the selected breast cancer cell lines were the aminosquaraine dyes **11** and **12** (Figure 4 and Figure 5). Contrary to what has been reported with HepG2 and Caco-2 cell lines [37,48], in which the introduction of amines in the four-membered central ring induced excessive cytotoxicity, and the introduction of zwitterionic dyes increased photodynamic activity, in our study the unsymmetrical squaraines showed the innocuousness of the unsubstituted squaraine dye and the selective photocytotoxicity of the aminosquaraine dyes for BT-474 and MCF-7 cells. The amines’ introduction can thus essentially lead to a significative rise in the cytotoxicity of this dye’s core; however, for the dyes presented in this study, it was found that this was intensified in a balanced way. Despite the evidence of a marked increase in cytotoxicity, which proved to be extremely advantageous in the present research, the amines’ introduction in this dye’s core is of great relevance, since, in addition to allowing an increase in its ability for producing singlet oxygen [77,78], as we verified on a small scale, it favorably increases its uptake by cells due to its cationic character and increases its polarity, thus favoring its interaction with biological systems [79].

Compared to other studies, namely two of our most recent studies involving breast cancer cell line MCF-7 and the potential application of indolenine-based aminosquaraine dyes [25,41], unsymmetrical squaraines present in this work showed similar levels of cytotoxicity in the presence of radiation, but more selective photocytotoxicity, since, for example, in non-irradiated conditions, *N*-hexyl indolenine-derived dyes only presented half inhibitory concentration values below 1 μM [25,41]. This dark cytotoxicity was subsequently improved by reducing the number of carbons in the *N*-alkyl chains of this dye’s core [25,41]. Nevertheless, it should be noted that, according to previous studies, dyes with longer carbon chains have longer wavelengths of maximum absorption and greater production of reactive oxygen species [80]. The improved cytotoxicity in the dark for MCF-7 metastatic cell line is also enhanced by the comparison with benzothiazole-derived symmetrical squaraine dyes, which showed half inhibitory concentrations in non-irradiated treatments below 0.6 μM [79]. Regarding the BT-474 cell line, to the best of our knowledge, until now there were no studies involving the photodynamic application of dyes analogous to those presented here.

Thus, in general, the aminosquaraine dyes synthesized in this work, despite having low weighting singlet oxygen quantum yields, showed worthy phototherapeutic effects for specific in vitro conditions. This photocytotoxicity, according to our experience, must come from type I reactions; still, studies focused on their action mechanism in the biological environment are ongoing. The moderate to good photostability, as well as the high absorption in the tissue transparency spectral region and photoselective cytotoxic action, make these compounds potential photosensitizer compounds to be applied in photodynamic therapy for breast cancer.

## 5. Conclusions

A series of quinoline- and benzoselenazole-derived squaraine and aminosquaraine cyanine dyes were designed, prepared, and characterized. All dyes showed intense bands in the region of the electromagnetic spectrum, where the living tissues are transparent to light. In organic solvents, the absorption bands, besides being intense, were narrow and had maximum absorption wavelengths between 636 and 733 nm and logarithms of the molar absorptivity coefficients around 5–6. In aqueous media, wider bands were observed due to the formation of aggregates. The constitution of aqueous media was found to influence the type of aggregates formed. Despite the forming of dimers in aqueous media, the absorptivity levels of these dyes remained high without compromising the potential photodynamic activity in a biological environment. The dyes proved to be light stable, highlighting the stability of the zwitterionic dye compared to the commercially available methylene blue photosensitizer. Nevertheless, this dye scaffold showed a low production of singlet oxygen. Despite this, their in vitro phototherapeutic activity was evident for specific conditions tested against the studied breast cancer cell lines. Of the evaluated dyes, the ones that stood out the most were the aminosquaraine dyes, which, in both cell lines, showed conditions in which the degree of cytotoxicity produced in the dark is much lower than when irradiated. The incubation periods with the dyes showed that these compounds have a prolonged activity over time, since there was a tendency to decrease cell viability, which was more accentuated in the period of 24 h compared to that of 1 h after being irradiated. Insignificant differences were observed between treatments irradiated for 7 and 14 min, proving that the shortest irradiation was sufficient to trigger their maximum biological activity. It is assumed that the dyes’ photobiological effects are mostly from type I reactions, as the reduced capacity of these dyes to produce singlet oxygen does not justify it by type II mechanism reactions.

## Figures and Tables

**Table 1:** Relevant proton and carbon nuclear magnetic resonance spectra signals of quinoline- and benzoselenazole-derived unsymmetrical squaraine cyanine dyes **9**–**12**.

Dye	^1^H NMR	^13^C NMR
	C=CH	C=CH
**9**	6.00 (1H, s)	92.85
	5.77 (1H, s)	88.91
**10**	6.19 (1H, s)	92.84
	5.75 (1H, s)	89.13
**11**	6.18 (1H, s)	93.89
	5.95 (1H, s)	89.15
**12**	6.15 (1H, s)	94.40
	6.07 (1H, s)	93.99
	5.92 (1H, s)	89.86
	5.85 (1H, s)	89.30

**Table 2:** Red and near-infrared spectroscopy data of quinoline- and benzoselanazole-derived unsymmetrical squaraine cyanine dyes **9**–**12** in organic solvents [acetonitrile (ACN), acetone (ACT), dichloromethane (DCM), dimethylformamide (DMF), dimethyl sulfoxide (DMSO), 1,4-dioxane (DXN), ethanol (EtOH), methanol (MeOH), tetrahydrofuran (THF), xylene (XLN)] and aqueous solvents [Dulbecco’s modified Eagle medium (DMEM), water and phosphate-buffered saline (PBS)]. Dyes’ maximum absorption wavelengths are presented in nm, and the logarithms of the molar absorptivity coefficients were determined by the application of the Lambert-Beet law in M^−1^·cm^−1^.

Solvent	Dye 9		Dye 10		Dye 11		Dye 12	
	λ_max_	log ε	λ_max_	log ε	λ_max_	log ε	λ_max_	log ε
**Organic Solvent**								
ACN	686	5.11	636	5.82	658	5.83	671	5.76
ACT	692	5.24	640	5.91	664	5.85	676	5.81
DCM	694	5.18	643	5.91	669	5.84	681	5.71
DMF	689	5.04	647	5.75	674	5.79	686	5.70
DMSO	700	5.20	651	5.82	679	5.80	690	5.75
DXN	700	5.15	655	5.73	680	5.74	689	5.69
EtOH	674	5.18	641	5.86	667	5.84	677	5.77
MeOH	690	5.28	661	5.86	684	5.81	695	5.76
THF	706	5.11	646	5.85	680	5.79	689	5.74
XLN	733	5.27	688	5.75	714	5.80	718	5.72
**Aqueous Solvent**								
DMEM	649	4.54	–	–	625	5.10	638	5.01
H_2_O	631	4.58	–	–	608	5.32	617	5.27
PBS	650	4.46	–	–	628	5.09	778	5.20

**Table 3:** Singlet oxygen quantum yield (Φ_Δ_) of quinoline- and benzoselenazole-derived unsymmetrical squaraine cyanine dyes **9**, **11** and **12** measured in chloroform (CFM) using phenazine (Φ_Δ_ = 0.84) as reference.

Φ_Δ_	Dye 9	Dye 11	Dye 12
CFM	0.03	0.04	0.06

**Table 4:** Estimated half-inhibitory concentration values (IC_50_ values, in µM) and coefficient of their determination (r^2^) for the quinoline- and benzoselenazole-derived unsymmetrical squaraine cyanine dyes **9**, **11** and **12** in BT-474 and MCF-7 cells exposed to 0, 7 or 14 min of light-emitting diode irradiation and 1 or 24 h of incubation with the irradiated dyes.

Dye	Irradiation Time	BT-474				MCF-7			
		1 h		24 h		1 h		24 h	
		IC_50_	r^2^	IC_50_	r^2^	IC_50_	r^2^	IC_50_	r^2^
**9**	0′	4.423	0.92	7.023	0.92	6.400	0.99	5.999	0.92
	7′	5.628	0.71	1.958	0.79	6.335	0.96	6.359	0.99
	14′	6.941	0.96	3.184	0.95	6.414	0.97	3.149	0.88
**11**	0′	1.209	0.99	0.706	0.99	4.018	0.98	1.225	0.98
	7′	0.203	0.99	0.207	0.99	0.313	0.98	0.189	0.99
	14’	0.197	0.99	0.097	0.99	0.140	0.99	0.084	0.99
**12**	0′	1.468	0.96	2.534	0.99	1.078	0.99	1.253	0.99
	7′	0.402	0.98	0.242	0.99	0.147	0.99	0.074	0.99
	14′	0.386	0.99	0.199	0.99	0.104	0.99	0.042	0.99

## Supplementary Materials

The following are available online at https://www.mdpi.com/1996-1944/13/11/2646/s1, Figure S1: ^1^H NMR spectrum of compound **5** (600 MHz, CDCl_3_, ppm). Residual solvent peaks: *Et_2_O, **CH_3_OH.; Figure S2: ^13^C NMR spectrum of compound **5** (150.90 MHz, CDCl_3_, ppm). Residual solvent peak: *Et_2_O.; Figure S3: HRESI-TOFMS spectrum of compound **5**.; Figure S4: ^1^H NMR spectrum of dye **9** (600 MHz, CDCl_3_, ppm). Residual solvent peak: *CH_3_OH.; Figure S5: ^13^C NMR spectrum of dye **9** (150.9 MHz, CDCl_3_, ppm); Figure S6: HRESI-TOFMS spectrum of dye **9**.; Figure S7: ^1^H NMR spectrum of dye **10** (400 MHz, CDCl_3_, ppm). Residual solvent peaks: *CH_2_Cl_2_, **CH_3_OH.; Figure S8: ^13^C NMR spectrum of dye **10** (150.9 MHz, CDCl_3_, ppm). Residual solvent peaks: *Et_2_O, **CH_2_Cl_2_.; Figure S9: LRESI-TOFMS spectrum of dye **10**.; Figure S10: ^1^H NMR spectrum of dye **11** (600 MHz, DMSO-*d*_6_, ppm); Figure S11: ^13^C NMR spectrum of dye **11** (150.9 MHz, DMSO-*d*_6_, ppm); Figure S12: LRESI-TOFMS spectrum of dye **11**.; Figure S13: ^1^H NMR spectrum of dye **12** (600 MHz, DMSO-*d*_6_, ppm). Residual solvent peak: *CH_2_Cl_2_.; Figure S14: ^13^C NMR spectrum of dye **12** (150.9 MHz, DMSO-*d*_6_, ppm). Residual solvent peak: *CH_2_Cl_2_.; Figure S15: LRESI-TOFMS spectrum of dye **12**.
