# Supplementary material for: Quinoline- and Benzoselenazole-Derived Unsymmetrical Squaraine Cyanine Dyes: Design, Synthesis, Photophysicochemical Features and Light-Triggerable Antiproliferative Effects against Breast Cancer Cell Lines
Source: Materials (Basel). 2020 Jun 10;13(11):2646. doi: 10.3390/ma13112646 (PMC7321604; doi:10.3390/ma13112646)
Supplement: Supplementary file 1 [file materials-13-02646-s001.pdf]

materials

Supplementary Materials

Quinoline- and Benzoselenazole-Derived Unsymmetrical Squaraine Cyanine Dyes: Design, Synthesis, Photophysicochemical Features and Light-Triggerable Antiproliferative Effects against Breast Cancer Cell Lines

Eurico Lima [1], Renato E. Boto [2], Diana Ferreira [3], José R. Fernandes [1], Paulo Almeida [2], Luis F. V. Ferreira [3], Eliana B. Souto [4,5], Amélia M. Silva [6,*] and Lucinda V. Reis [1,*]

[1] Chemistry Centre of Vila Real (CQ-VR), University of Trás-os-Montes and Alto Douro, Quinta de Prados, 5001-801 Vila Real, Portugal; eurico_lima@icloud.com (E.L.); jraf@utad.pt (J.R.F.)
[2] Health Sciences Research Centre (CICS-UBI), University of Beira Interior, Av. Infante D. Henrique, 6201-001 Covilhã, Portugal; rboto@ubi.pt (R.E.B.); pjsa@ubi.pt (P.A.)
[3] Institute of Bioengineering and Biosciences (iBB), Higher Technical Institute, University of Lisbon, Av. Rovisco Pais, 1049-001 Lisbon, Portugal; diana.ferreira@det.uminho.pt (D.F.); luisfilipevf@ist.utl.pt (L.F.V.F.)
[4] Department of Pharmaceutical Technology, Faculty of Pharmacy, University of Coimbra, Pólo das Ciências da Saúde, Azinhaga de Santa Comba, 3000-548 Coimbra, Portugal; ebsouto@ff.uc.pt
[5] Centre of Biological Engineering (CEB), University of Minho, Campus de Gualtar, 4710-057 Braga, Portugal
[6] Department of Biology and Environment (DeBA), and Centre for Research and Technology of Agro-Environmental and Biological Sciences (CITAB-UTAD), University of Trás-os-Montes and Alto Douro, Quinta de Prados, 5001-801 Vila Real, Portugal
* Correspondence: amsilva@utad.pt (A.M.S.); lucinda.reis@utad.pt (L.V.R.)

Received: 30 April 2020; Accepted: 8 June 2020; Published: date

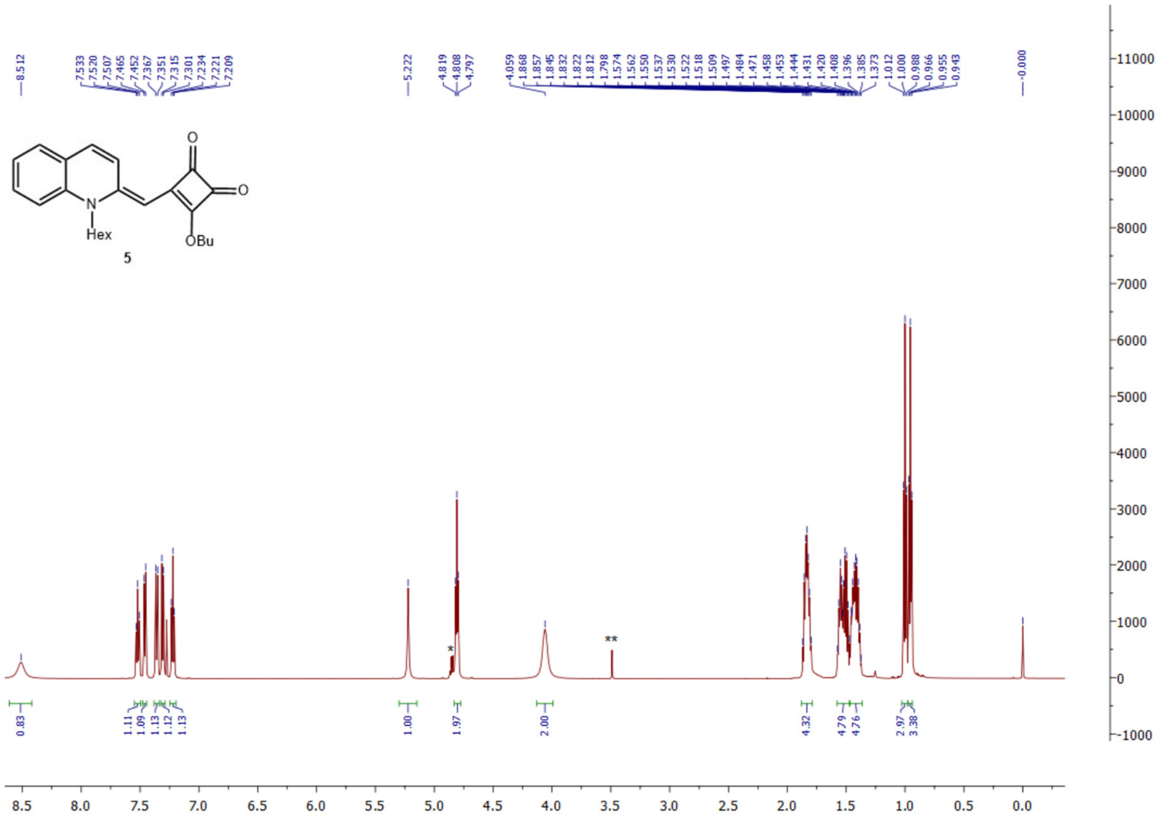

Figure S1. NMR spectrum of compound **5** (600 MHz, $CDCl_3$, ppm). Residual solvent peaks: *Et_2O, **CH_3OH.

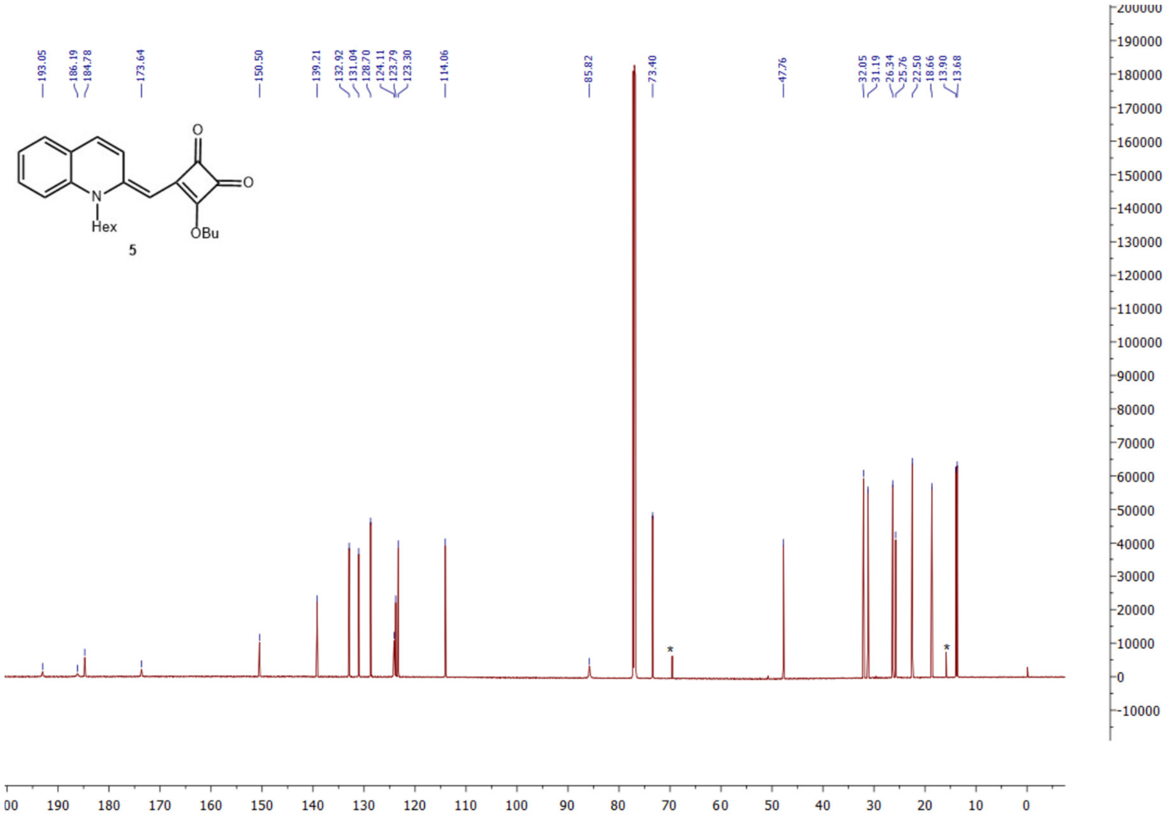

Figure S2 ^{13}C NMR spectrum of compound **5** (150.90 MHz, CDCl3, ppm). Residual solvent peak*:* *Et_2O.

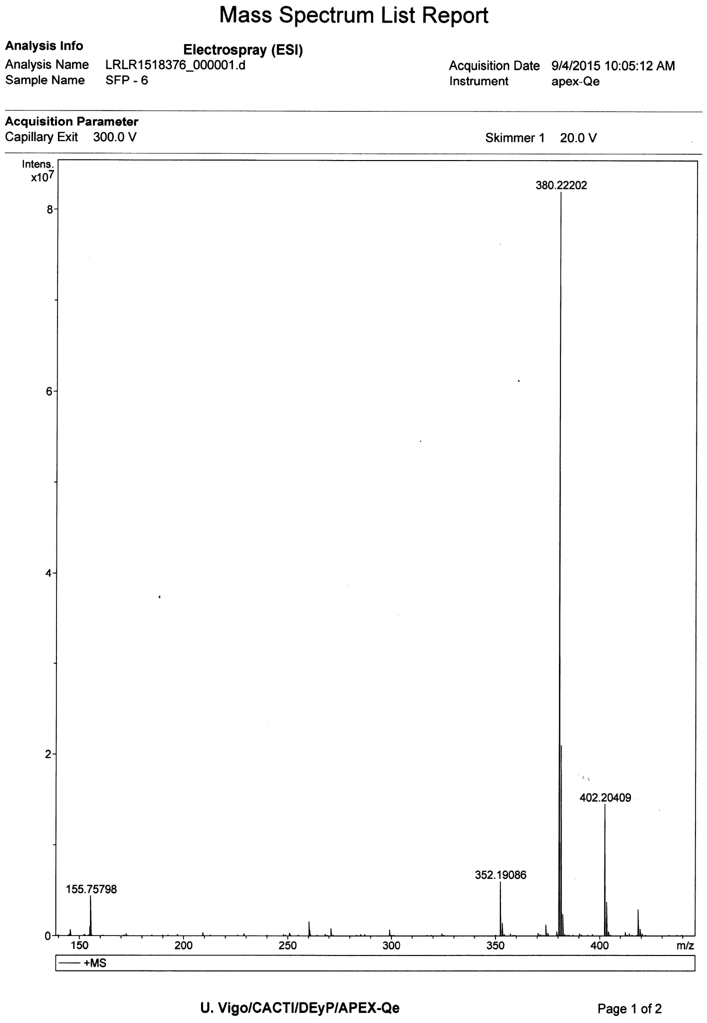

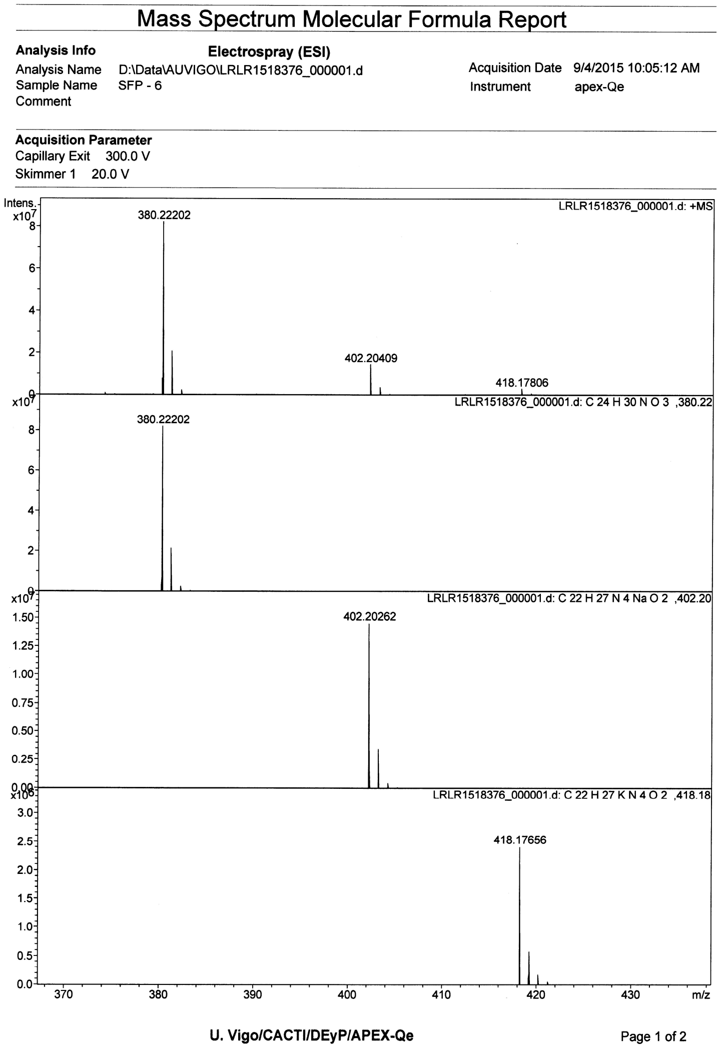

Figure S3. HRESI-TOFMS spectrum of compound **5**.

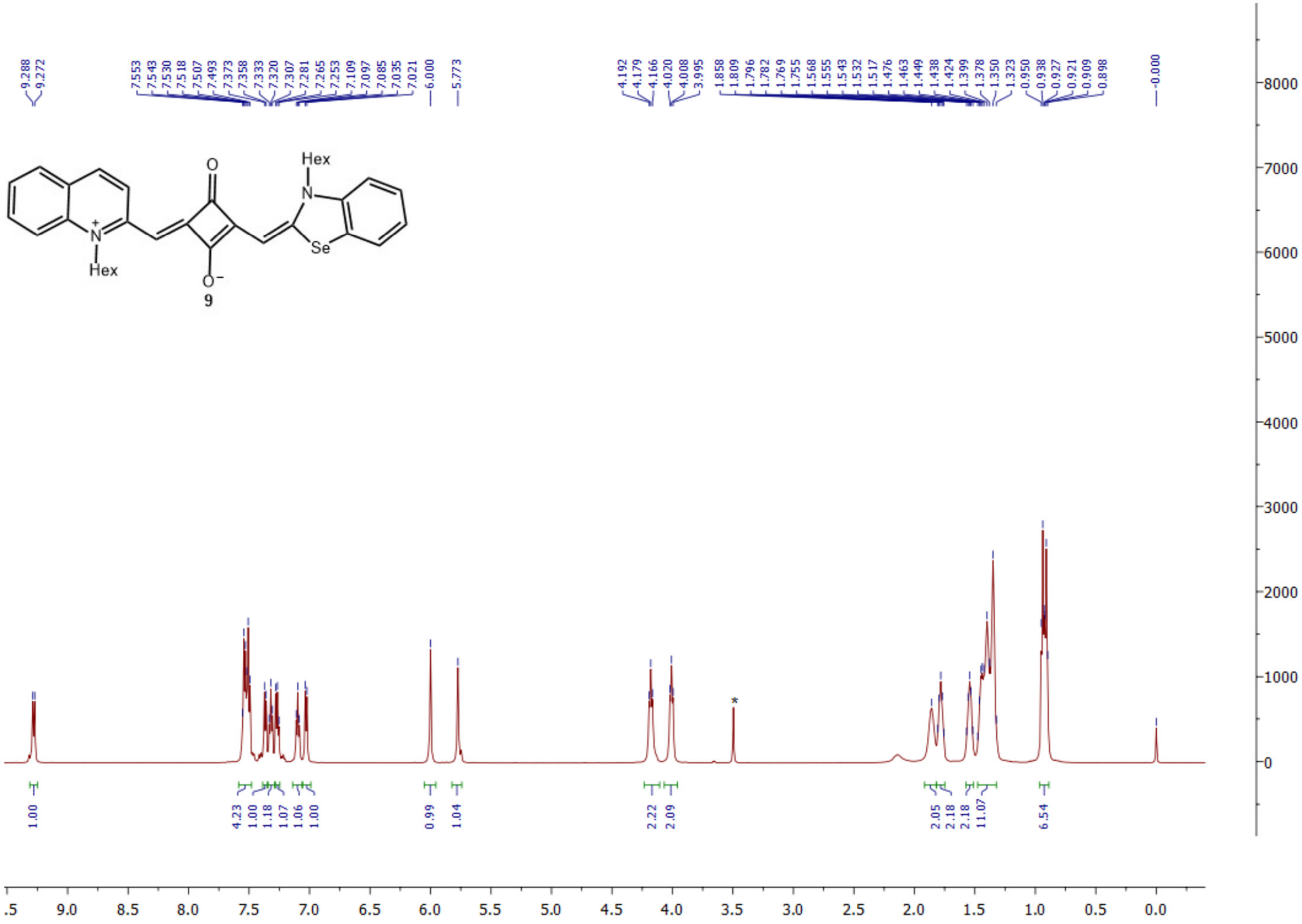

Figure S4 ^{1}H NMR spectrum of dye **9** (600 MHz, $CDCl_3$, ppm). Residual solvent peak: *CH_3OH.

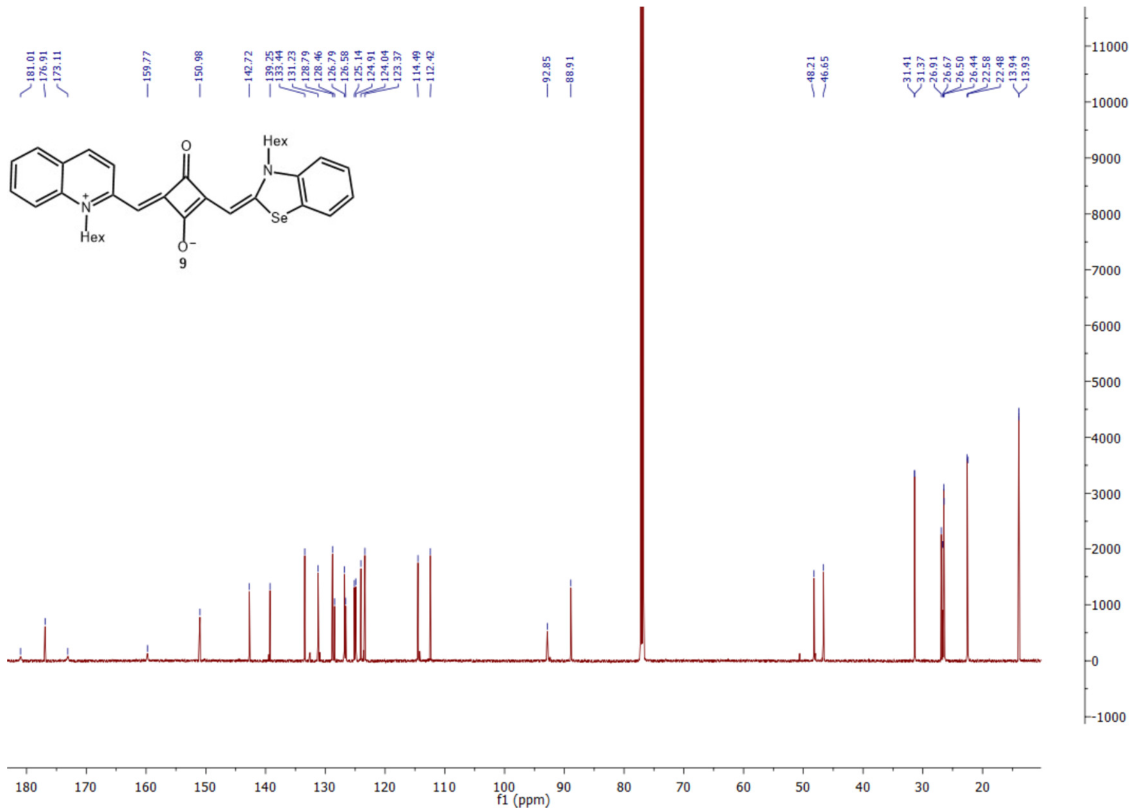

Figure S5. ^{13}C NMR spectrum of dye **9** (150.90 MHz, $CDCl_3$, ppm).

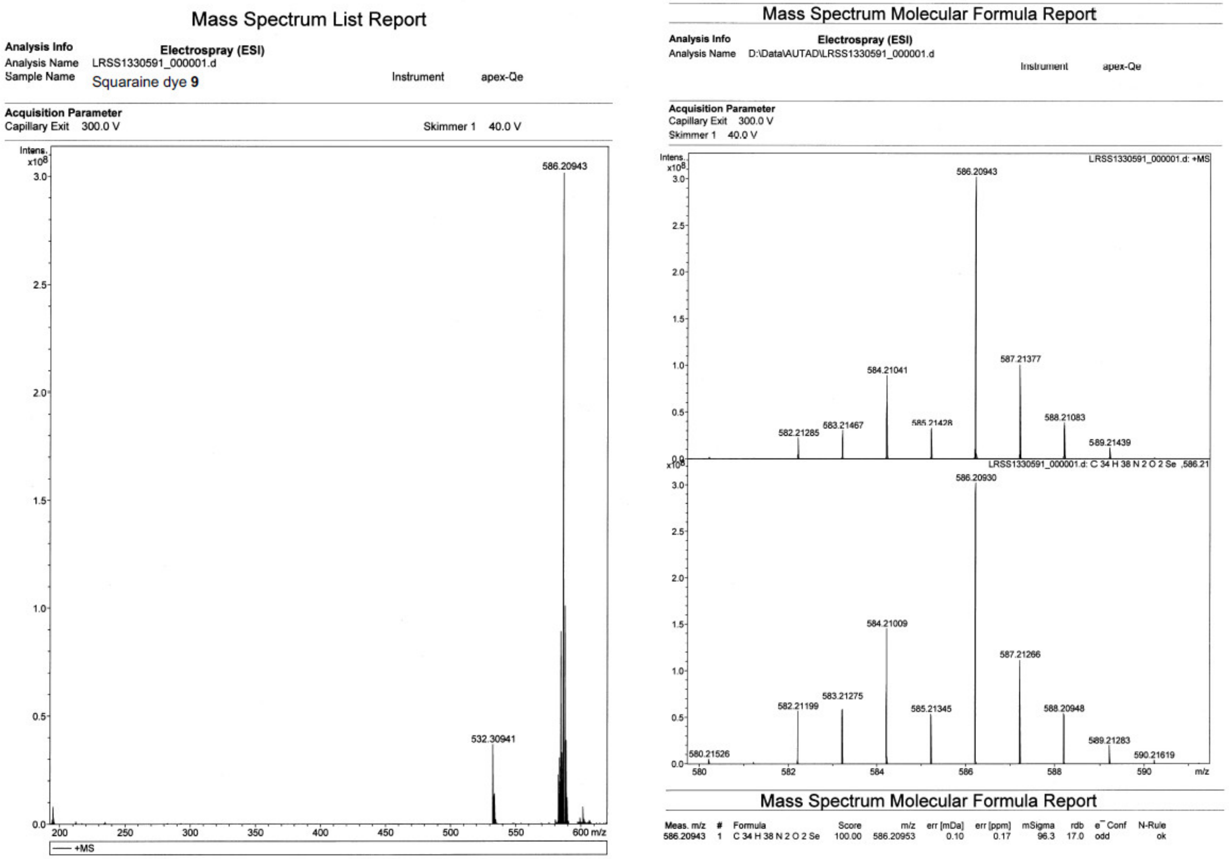

Figure S6. HRESI-TOFMS spectrum of dye **9**.

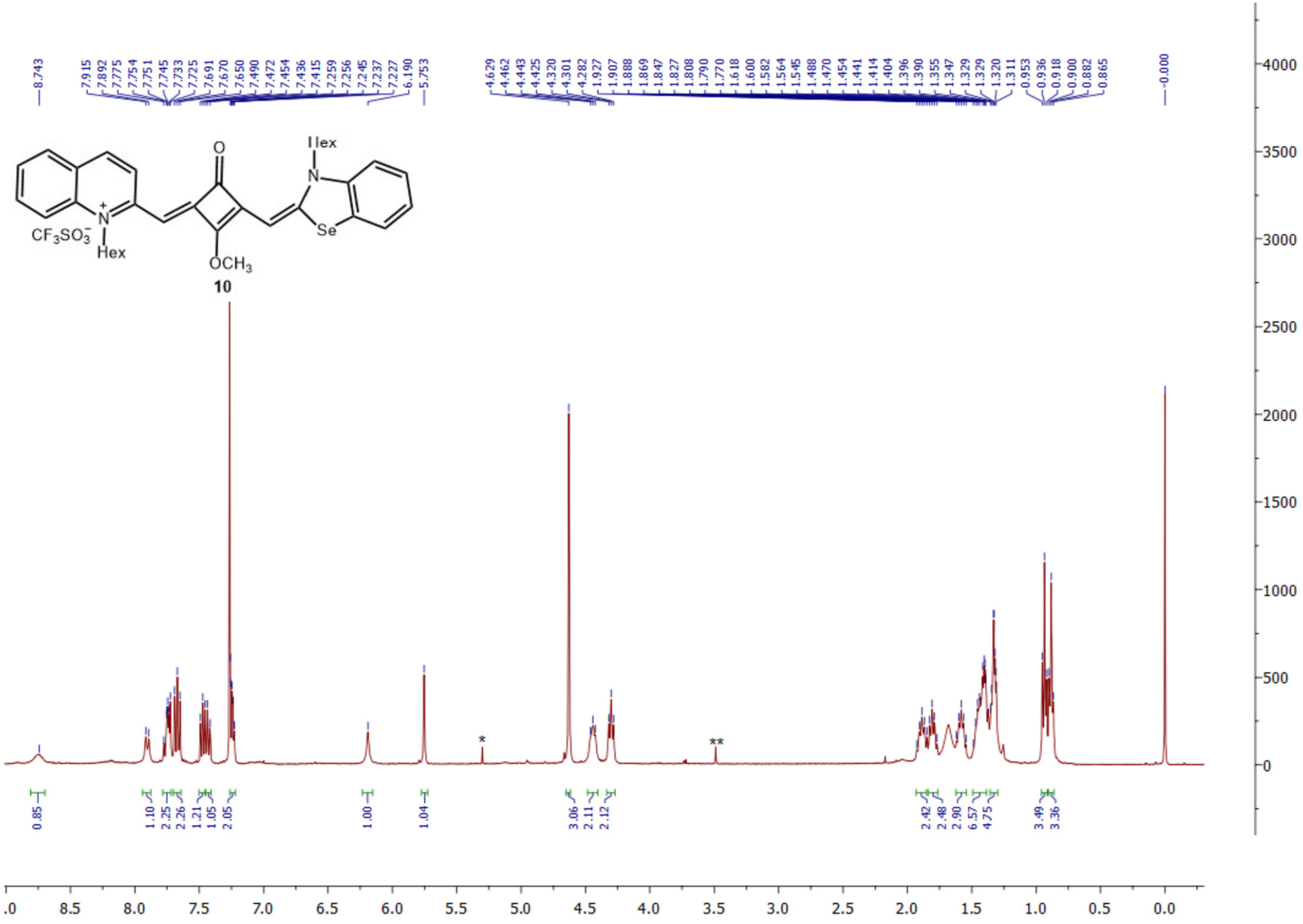

Figure S7 ^{1}H NMR spectrum of dye **10** (400 MHz, $CDCl_3$, ppm). Residual solvent peaks: *CH_2Cl_2, **CH_3OH.

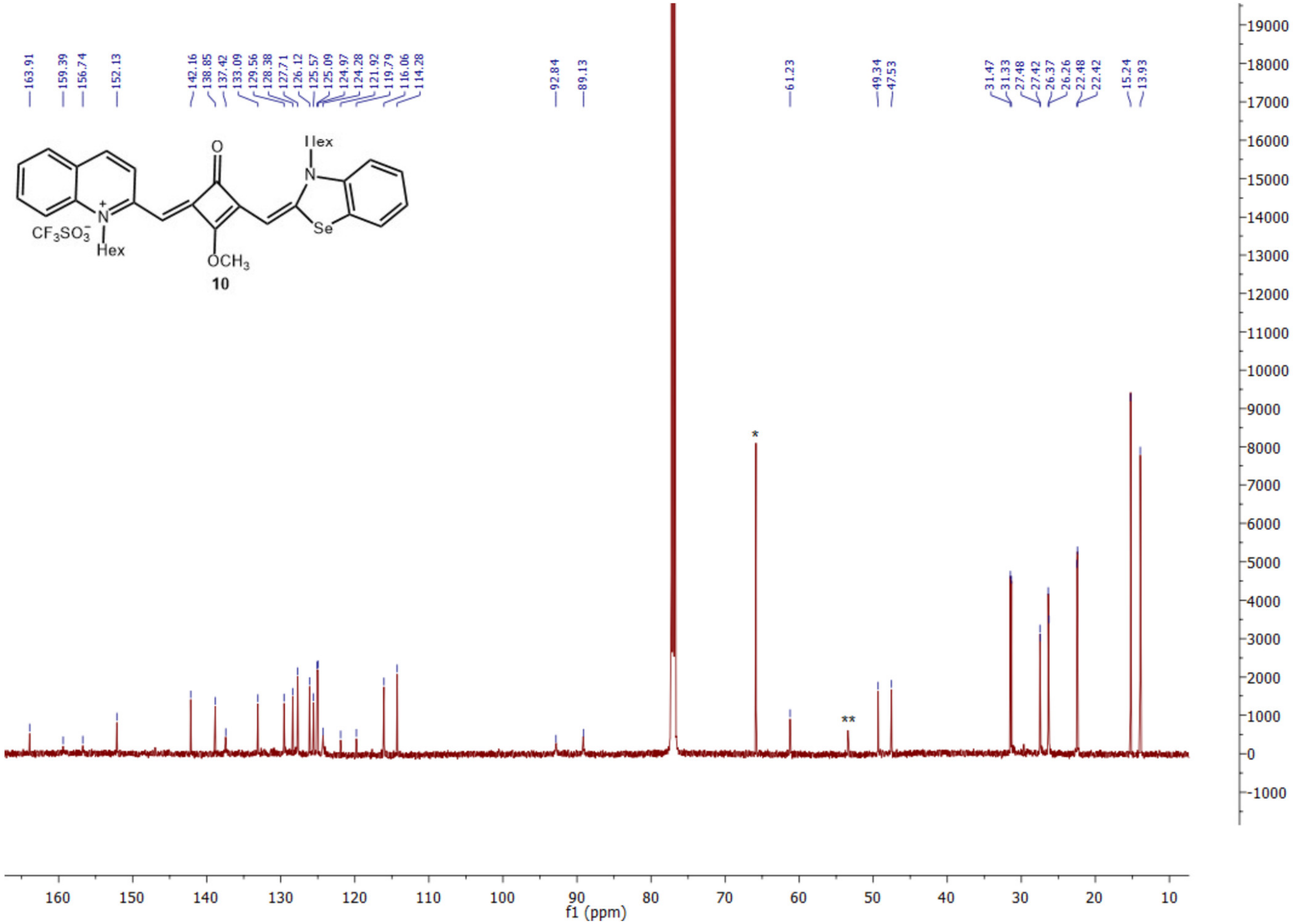

Figure S8 ^{13}C NMR spectrum of dye **10** (150.90 MHz, $CDCl_3$, ppm). Residual solvent peaks: *Et_2O, **CH_2Cl_2.

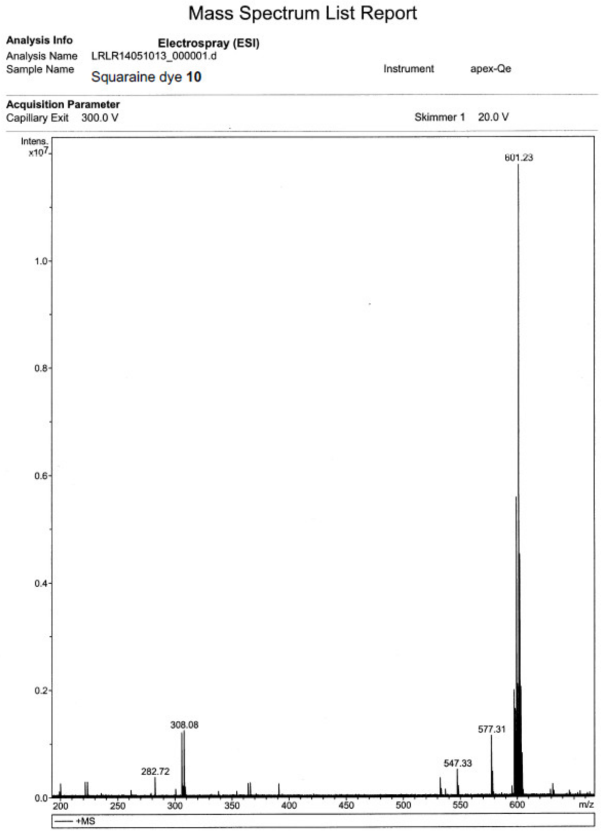

Mass Spectrum List Report

m/z	I	Res.
282.72	388779	227875
306.09	1214255	204184
308.08	1251342	203710
547.33	521700	115038
577.31	1149126	109288
578.31	482512	111303
597.23	2016358	105716
598.24	1653287	97591
599.23	650920	181766
599.23	5608072	104244
600.24	2122281	105640
601.23	11813129	105370
601.24	611369	204783
602.24	4545482	104442
603.23	2077289	98418
603.24	637425	83756
604.24	833224	102542

Figure S9. LRESI-TOFMS spectrum of dye **10**.

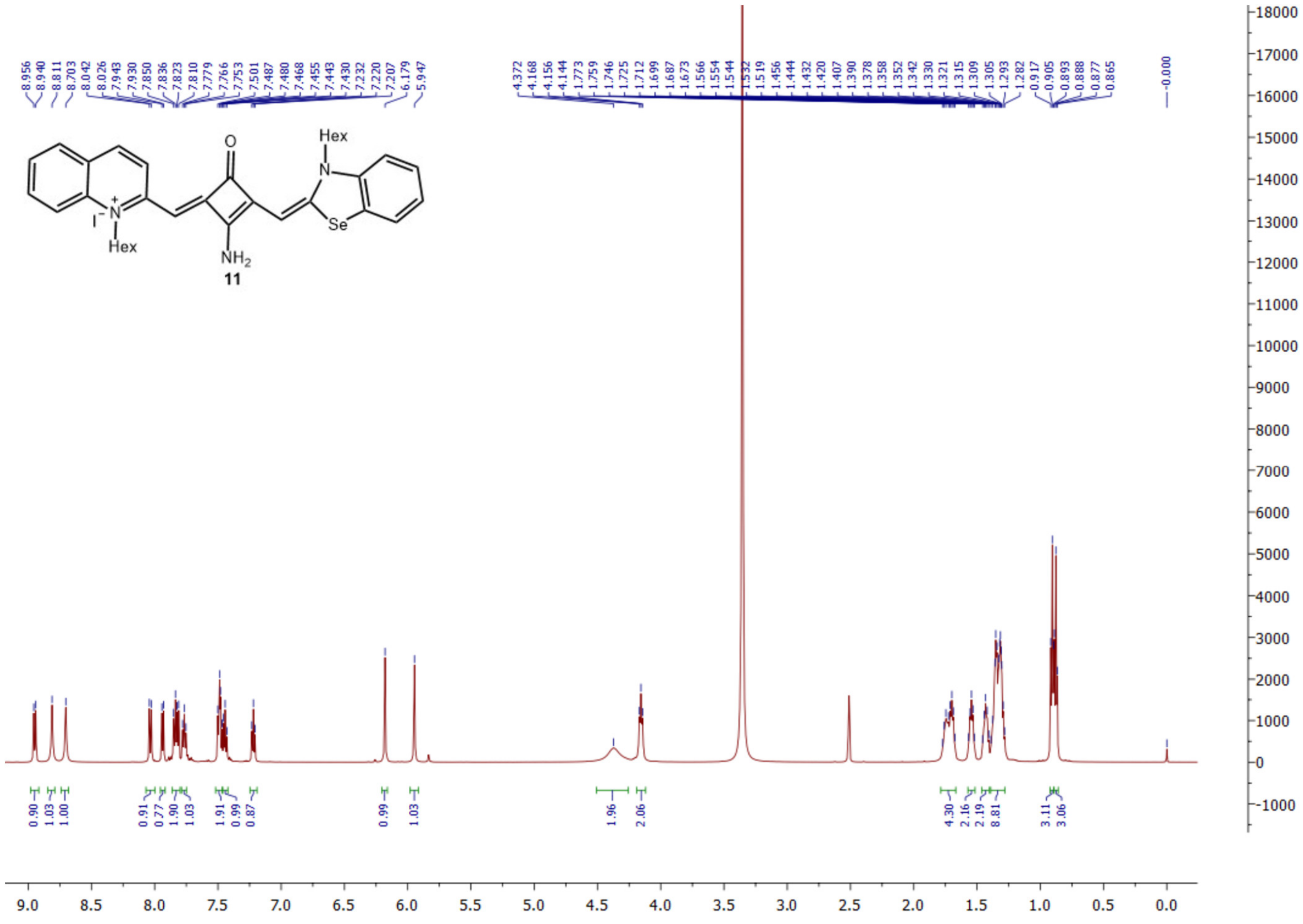

Figure S10 ^{1}H NMR spectrum of dye **11** (600 MHz, DMSO-d_6, ppm).

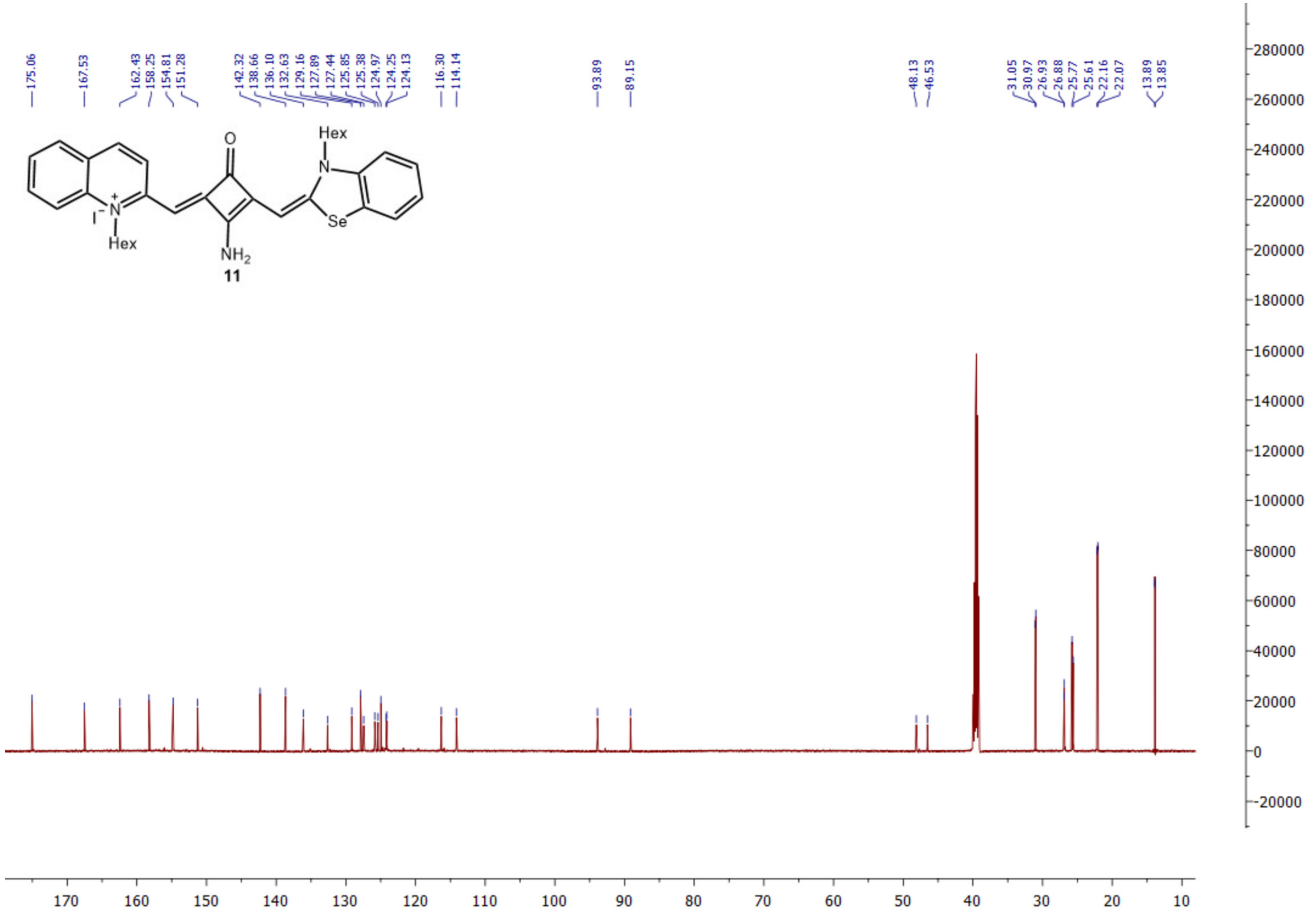

Figure S11 ^{13}C NMR spectrum of dye **11** (150.90 MHz, DMSO-d$_6$, ppm).

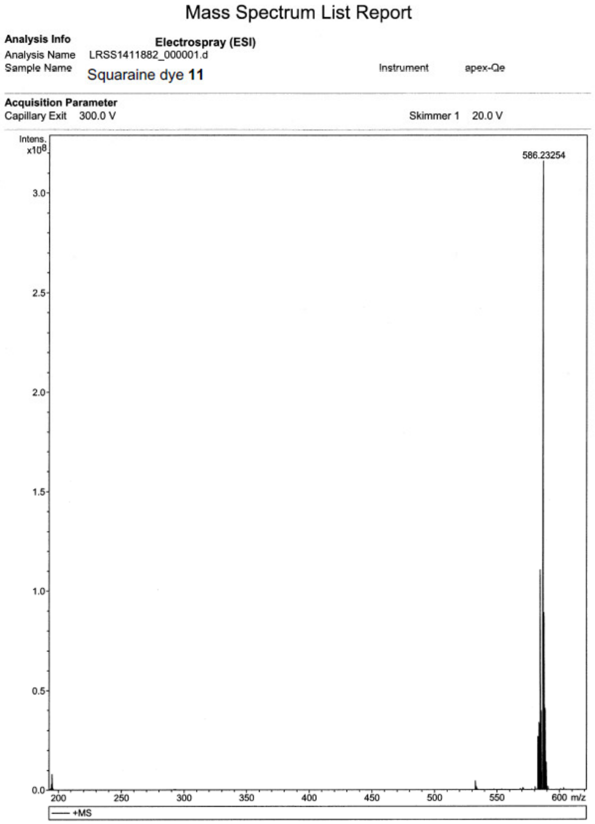

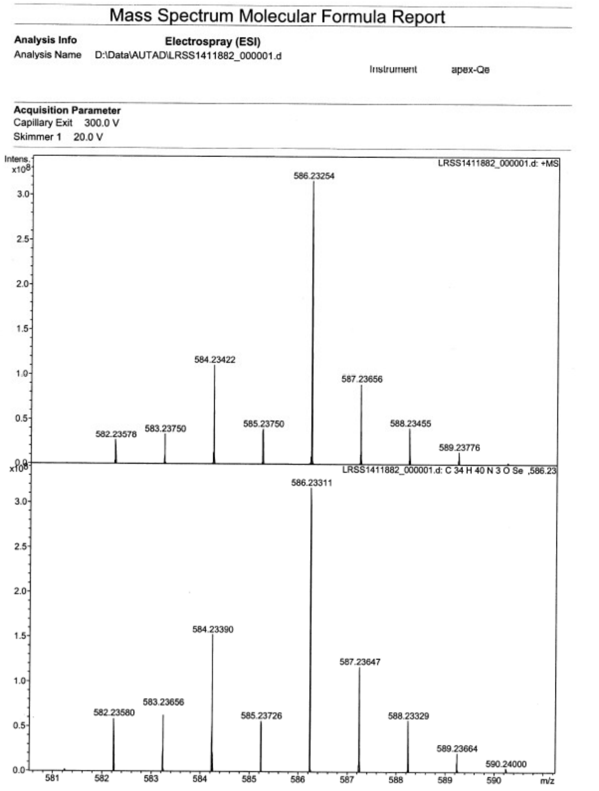

Mass Spectrum Molecular Formula Report

Meas. m/z	#	Formula	Score	m/z	err [mDa]	err [ppm]	mSigma	rdb	e⁻ Conf	N-Rule
586.23254	1	C 34 H 40 N 3 O Se	100.00	586.23334	0.57	0.97	91.0	16.5	even	ok
	2	C 36 H 42 O 2 Se	36.19	586.23471	1.91	3.26	93.5	16.0	odd	ok

Figure S12. HRESI-TOFMS spectrum of dye **11**.

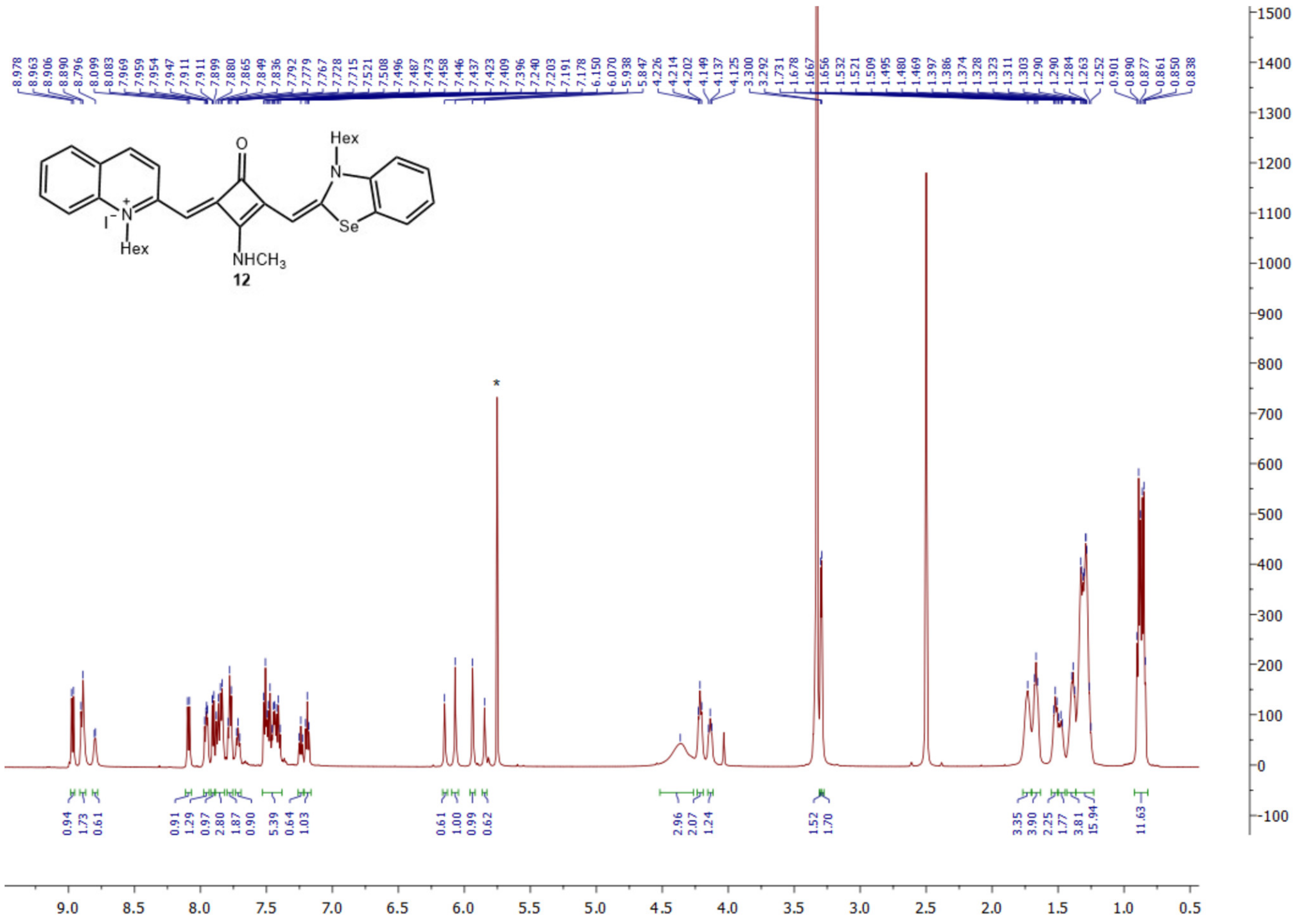

Figure S13. NMR spectrum of dye **12** (600 MHz, DMSO-d6, ppm). Residual solvent peak: *CH_2Cl_2.

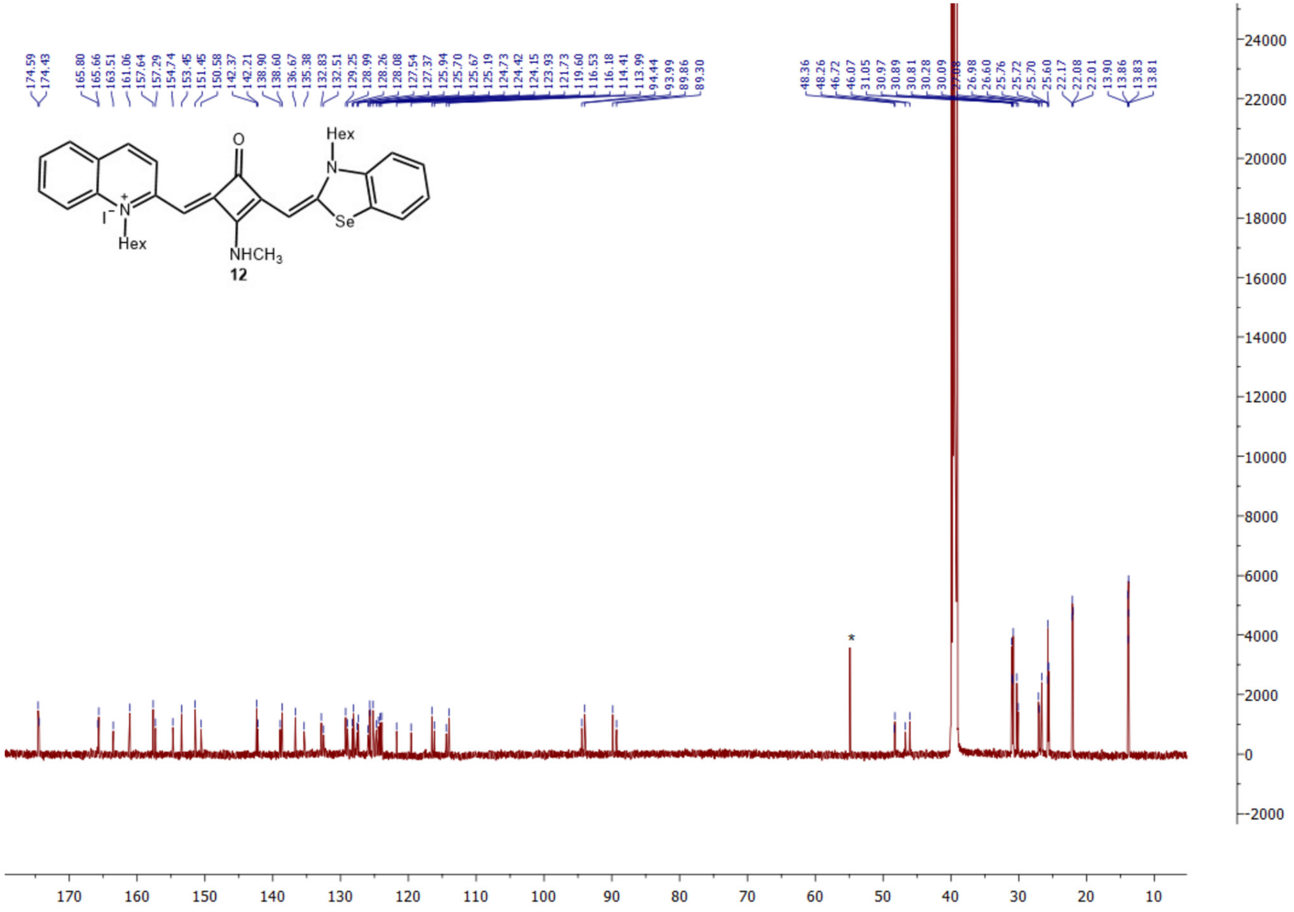

Figure S14. ^{13}C NMR spectrum of dye **12** (150.90 MHz, DMSO-d_6, ppm). Residual solvent peak: *CH_2Cl_2.

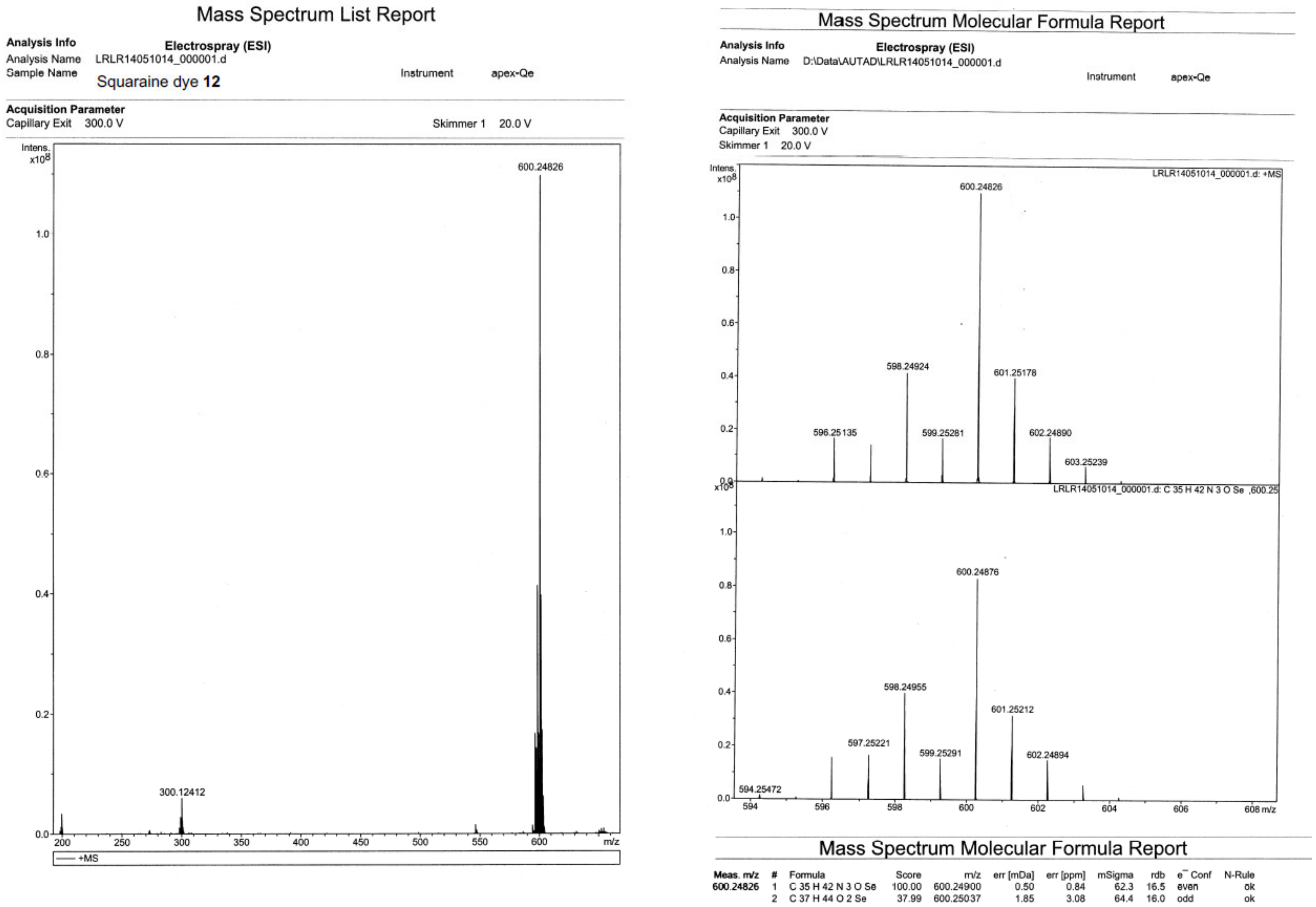

Figure S15. HRESI-TOFMS spectrum of dye **12**.
